# Stochastic approximation cut algorithm for inference in modularized Bayesian models

**DOI:** 10.1007/s11222-021-10070-2

**Published:** 2021-12-06

**Authors:** Yang Liu, Robert J. B. Goudie

**Affiliations:** 1 MRC Biostatistics Unit, University of Cambridge, Cambridge, UK

**Keywords:** Cutting feedback, Stochastic approximation Monte Carlo, Intractable normalizing functions, Discretization

## Abstract

Bayesian modelling enables us to accommodate complex forms of data and make a comprehensive inference, but the effect of partial misspecification of the model is a concern. One approach in this setting is to modularize the model and prevent feedback from suspect modules, using a cut model. After observing data, this leads to the cut distribution which normally does not have a closed form. Previous studies have proposed algorithms to sample from this distribution, but these algorithms have unclear theoretical convergence properties. To address this, we propose a new algorithm called the stochastic approximation cut (SACut) algorithm as an alternative. The algorithm is divided into two parallel chains. The main chain targets an approximation to the cut distribution; the auxiliary chain is used to form an adaptive proposal distribution for the main chain. We prove convergence of the samples drawn by the proposed algorithm and present the exact limit. Although SACut is biased, since the main chain does not target the exact cut distribution, we prove this bias can be reduced geometrically by increasing a user-chosen tuning parameter. In addition, parallel computing can be easily adopted for SACut, which greatly reduces computation time.

## 1 Introduction

Bayesian models mathematically formulate our beliefs about the data and parameter. Such models are often highly structured models that represent strong assumptions. Many of the desirable properties of Bayesian inference require the model to be correctly specified. We say a set of models *f* (*x* |*θ*), where *θ* ∈ *Θ* , are misspecified if there is no *θ*_0_ ∈ *Θ* such that data *X* is independently and identically generated from *f* (*x* |*θ*_0_) (Walker 2013). In practice, models will inevitably fall short of covering every nuance of the truth. One popular approach when a model is misspecified is fractional (or power) likelihood. This can be used in both classical (e.g. Nakaya et al. 2005; Huang et al. 2010; Liu et al. 2018) and Bayesian (e.g. Miller and Dunson 2019; Bhattacharya et al. 2019) frameworks. However, this method treats all of the models as equally misspecified.

We consider the situation when the assumptions of the model are thought to partially hold: specifically, we assume that one distinct component (or module in the terminology of Liu et al. 2009) is thought to be incorrectly specified, whereas the other component is correctly specified. In standard Bayesian inference, these distinct modules are linked by Bayes’ theorem. Unfortunately, this means the reliability of the whole model may be affected even if only one component is incorrectly specified. To address this, in this paper we adopt the idea of “cutting feedback” (Lunn et al. 2009b; Liu et al. 2009; Plummer 2015; Jacob et al. 2017, 2020) which modifies the links between modules so that estimation of non-suspect modules is unaffected by information from suspect modules. This idea has been used in a broad range of applications including the study of population pharmacokinetic/pharmacodynamic (PK/PD) models (Lunn et al. 2009a), analysis of computer models (Liu et al. 2009), Bayesian estimation of causal effects with propensity scores (McCandless et al. 2010; Zigler 2016) and Bayesian analysis of health effect of air pollution (Blangiardo et al. 2011).

**Fig. 1:**
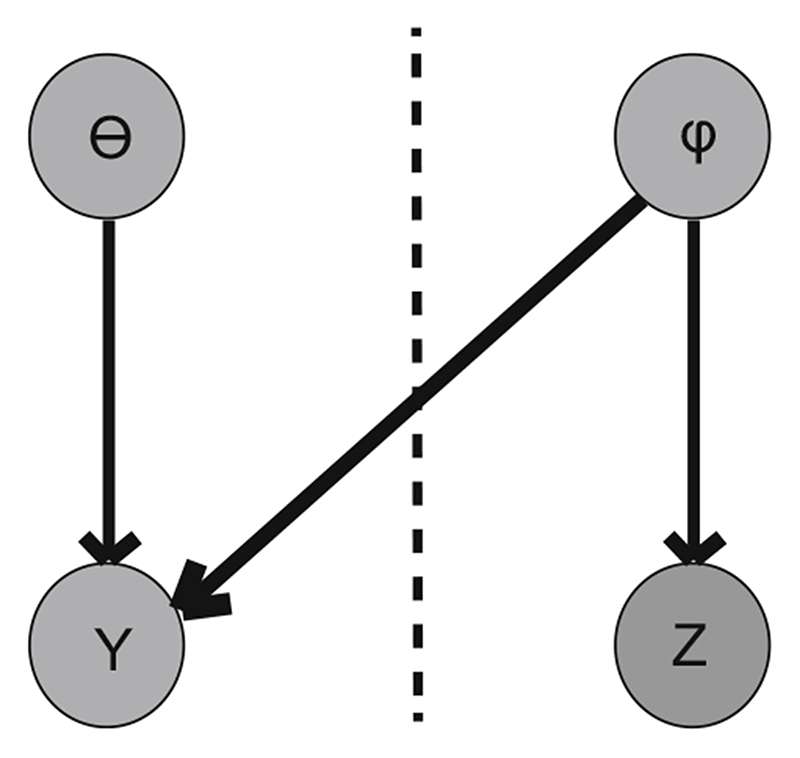
DAG representation of a generic two-module model. The two modules are separated by a dashed line

Consider the generic two-module model with observable quantities (data) *Y* and *Z* and parameters *θ* and *φ*, shown in the directed acyclic graph (DAG) in Fig. 1. The joint distribution is

p(Y,Z,θ,φ)=p(Y∣θ,φ)p(Z∣φ)p(θ)p(φ),

and the standard Bayesian posterior, given observations of *Y* and *Z*, is

$$\begin{array}{lll} p(\theta ,\varphi |Y,Z) & \text{=} & p(\theta |Y,\varphi )p(\varphi |Y,Z)\\ \; & \text{=} & \frac{p(Y|\theta ,\varphi )p(\theta )}{p(Y|\varphi )}\frac{p(Y|\varphi )p(Z|\varphi )p(\varphi )}{p(Y,Z)}. \end{array}$$

Suppose we are confident that the relationship between *φ* and *Z* is correctly specified but not confident about the relationship between *φ* and *Y*. To prevent this possible misspecification affecting estimation of *φ*, we can “cut” feedback by replacing *p*(*φ*|*Y*,*Z*) in the standard posterior with *p*(*φ*|*Z*), making the assumption that *φ* should be solely estimated by *Z*,

(1) $$\begin{array}{lll} p_{\text{cut}}(\theta ,\varphi ) & \text{:=} & p(\theta |Y,\varphi )p(\varphi |Z)\\ \; & \text{=} & \frac{p(Y|\theta ,\varphi )p(\theta )}{p(Y|\varphi )}\frac{p(Z|\varphi )p(\varphi )}{p(Z)}. \end{array}$$

We call (1) the “cut distribution”. The basic idea of cutting feedback is to allow information to “flow” in the direction of the directed edge, but not in the reverse direction (i.e. a “valve” is added to the directed edge).

Sampling directly from *p*_cut_(*θ*, *φ*) is difficult because the marginal likelihood *p*(*Y* |*φ*) = ∫ *p*(*Y* |*θ*,*φ*)*p*(*θ*)d*θ* depends on a parameter of interest *φ* and is not usually analytically tractable, except in the simple case when *p*(*θ*) is conditionally conjugate to *p*(*Y* |*θ*, *φ*), which we do not wish to assume. This intractable marginal likelihood is a conditional posterior normalizing constant: it is the normalizing function for the posterior distribution *p*(*θ*|*Y*,*φ*), conditional on *φ*, of a parameter *θ* of interest:

(2) $$p(\theta |Y,\varphi )=\frac{p(Y,\theta |\varphi )}{p(Y|\varphi )}.$$

This differs importantly to intractable likelihood normalizing constants, as discussed in the doubly intractable literature (e.g. Park and Haran 2018), in which the normalizing function *H*(*φ*)= ∫*h*(*Y*|*φ*)*dY* for the likelihood is intractable.

$$p(Y|\varphi )=\frac{h(Y|\varphi )}{H(\varphi )}.$$

The normalizing function *H* (*φ*) is obtained by marginalizing the likelihood, with respect to the observable quantity *Y*, in contrast to the normalizing function *p*(*Y* |*φ*), which is obtained by marginalizing likelihood *p*(*Y*,*θ*|*φ*) with respect to a parameter *θ* of interest. This difference means that standard methods for doubly intractable problems (e.g. Møller et al. 2006; Murray et al. 2006; Liang 2010; Liang et al. 2016), which introduce an auxiliary variable, with the same distribution (or proposal distribution) as the distribution of the *a posteriori* observed and fixed *Y* to cancel the intractable normalizing function shared by them, do not directly apply to (2).

A simple algorithm that aims to draw samples from *p*_cut_(*θ*, *φ*) is implemented in WinBUGS (Lunn et al. 2009b). It is aGibbs-style sampler that involves updating *θ* and *φ* with a pair of transition kernels $q\left(\theta^{\prime }|\theta ,\varphi^{\prime }\right)$ and $q\left(\varphi^{\prime }|\varphi \right)$ that satisfy detailed balance with $p\left(\theta |Y,\varphi^{\prime }\right)$ and *p*(*φ*|*Z*), respectively. However, the chain constructed by theWinBUGS algorithm may not have the cut distribution as its stationary distribution (Plummer 2015) since

$$\int p_{\text{cut}}(\theta ,\varphi )q\left(\theta^{\prime }|\theta ,\varphi^{\prime }\right)q\left(\varphi^{\prime }|\varphi \right)\text{d}\theta \text{d}\varphi =w\left(\theta^{\prime },\varphi^{\prime }\right)p_{\text{cut}}\left(\theta^{\prime },\varphi^{\prime }\right),$$

where the weight function *w* is

$$w\left(\theta^{\prime },\varphi^{\prime }\right)=\int \frac{p(\theta |Y,\varphi )}{p\left(\theta |Y,\varphi^{\prime }\right)}q\left(\varphi |\varphi^{\prime }\right)q\left(\theta |\theta^{\prime },\varphi^{\prime }\right)\text{d}\theta \text{d}\varphi .$$

The WinBUGS algorithm is inexact since $w\left(\theta^{\prime },\varphi^{\prime }\right)\neq 1$ , except in the simple case (conditionally conjugate) when it is possible to draw exact Gibbs updates from $p\left(\theta^{\prime }|Y,\varphi^{\prime }\right)$ . Plummer (2015) proposed two algorithms that address this problem by satisfying $w\left(\theta^{\prime },\varphi^{\prime }\right)=1$ approximately. One is a nested MCMC algorithm, which updates *θ* from $p\left(\theta^{\prime }|Y,\varphi^{\prime }\right)$ by running a separate internal Markov chain with transition kernel $q^{*}\left(\theta^{\prime }|\theta ,\varphi^{\prime }\right)$ satisfying detailed balance with the target distribution $p\left(\theta |Y,\varphi^{\prime }\right)$ . The other is a linear path algorithm, which decomposes the complete MCMC move from (*θ*, *φ*) to $\left(\theta^{\prime },\varphi^{\prime }\right)$ into a series of substeps along a linear path from *φ* to $\varphi^{\prime }$ and drawing a new *θ* at each substep. However, these methods require either the length of the internal chain or the number of substeps to go to infinity, meaning that in practice, these algorithms will not necessarily converge to *p*_cut_.

**Fig. 2:**
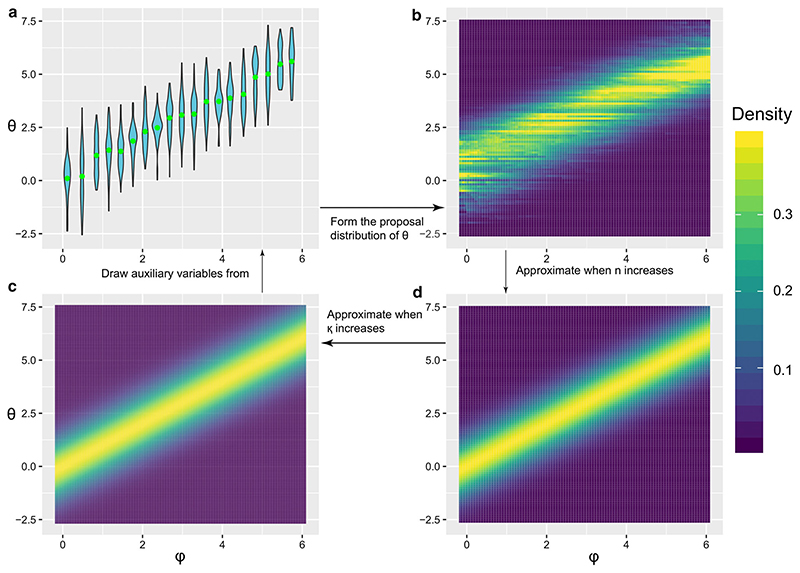
Relationship between $p\left(\theta |Y,\varphi_{0}^{\left(i\right)}\right),p(\theta |Y,\varphi ),p^{\left(\kappa \right)}(\theta |Y,\varphi )$ and $p_{n}^{\left(\kappa \right)}(\theta |Y,\varphi )$ . This is a toy example when the conditional distribution of *θ*, given *Y* = 1 and *φ*, is N(*φ*,*Y* ^2^). Samples of the auxiliary variable $\tilde{\theta }$ are drawn from a mixture of discretized densities $p\left(\theta |Y,\varphi_{0}^{\left(i\right)}\right)$ , *i* = 1,…,*m*, shown in the violin plot in **a**, with the green dots showing the median of each component (see Sect.2.1). Then $p_{n}^{\left(\kappa \right)}(\theta |Y,\varphi )$ , shown in **b**, is formed by using these auxiliary variables (see Sect. 2.2). Lemma 1 (Sect. 2.3) shows that $p_{n}^{\left(\kappa \right)}(\theta |Y,\varphi )$ converges to *p* ^(κ)^(*θ*|*Y*,*φ*), which is shown in **d**, while (8) shows that *p* ^(κ)^(*θ*|*Y*,*φ*) converges to the original density *p*(*θ*|*Y*, *φ*), shown in **c**

In this article, we propose a new sampling algorithm for *p*_cut_(*θ*, *φ*), called the stochastic approximation cut (SACut) algorithm. Since *φ* can be easily sampled from tractable part *p*(*φ*|*Z*), our algorithm aims to sample *θ* from the intractable part *p*(*θ*|*Y*,*φ*). Our algorithm is divided into two chains that are run in parallel: the main chain that approximately targets *p*_cut_(*θ*,*φ*) and an auxiliary chain that is used to form a proposal distribution for *θ*|*φ* in the main chain (Fig. 2b). The auxiliary chain uses stochastic approximation Monte Carlo (SAMC) (Liang et al. 2007) to approximate the intractable marginal likelihood *p*(*Y* |*φ*) and subsequently form a discrete set of distribution *p*(*θ*|*Y*,*φ*) for each $\varphi \in {\text{Φ}}_{0}=\left\{\varphi_{0}^{\left(1\right)},\ldots ,\varphi_{0}^{\left(m\right)}\right\}$ , a set of pre-selected auxiliary parameters (Fig. 2a).

The basic “naive” form of our algorithm has convergence in distribution, but stronger convergence properties can be obtained by building a proposal distribution $p_{n}^{\left(\kappa \right)}(\theta |Y,\varphi )$ to target an approximation *p* ^(κ)^(*θ*|*Y*,*φ*) instead of the true distribution *p*(*θ*|*Y*,*φ*) (Fig. 2c, d). We prove a weak law of large numbers for the samples ${\left\{\left(\theta_{n},\varphi_{n}\right)\right\}}_{n=1}^{N}$ drawn from the main chain. We also prove that the bias due to targeting *p* ^(κ)^(*θ*|*Y*,*φ*) can be controlled by the precision parameter κ, and that the bias decreases geometrically as κ increases. Our algorithm is inspired by the adaptive exchange algorithm (Liang et al. 2016), but replaces the exchange step with a direct proposal distribution for *θ* given *φ* in the main chain.

## 2 Main result

Let the product space *Θ*×Φ be the supports of *θ* and *φ* under *p*_cut_. We assume the following throughout for simplicity.

**Assumption 1** (a) *Θ* and Φ are compact and (b) *p*_cut_ is continuous with respect to *θ* and *φ* over *Θ* × Φ.

Assumption 1(a) is restrictive, but is commonly assumed in the study of adaptive Markov chains (Haario et al. 2001). In most applied settings, it is reasonable to choose a prior for *θ* and *φ* with support on only a compact domain, making the domain of the cut distribution compact without any alteration to the likelihood. Note that Assumption 1 implies that *p*_cut_ is bounded over *Θ* × Φ. From now on, define a probability space (Ω, 𝓕, ℙ). Denote Lebesgue measure μ on *Θ* and Φ and let *p*_cut_ be the measure on *Θ* × Φ defined by its density *p*_cut_.

In following sections, we describe the construction of the algorithm. The naive version of our algorithm builds a discrete proposal distribution for *θ*, based on Liang et al. (2016). Note that, in Liang et al. (2016), this proposal distribution only draws auxiliary variables that are discarded once the parameter of interest is drawn, and so strong convergence results for samples drawn by this probability distribution are not needed by Liang et al. (2016). This does not always apply to our problem since *θ* is the parameter of interest and its parameter space can be continuous. The naive version does not allow us to prove stronger convergence and theoretical properties, so we apply a simple function approximation technique with a specially designed partition of the parameter space that enables a straightforward implementation. Although this approximation leads to bias, we showthat it can be controlled.

### 2.1 Naive stochastic approximation cut algorithm

To introduce ideas that we will use in Sect. 2.3, we first describe a naive version of the stochastic approximation cut algorithm. The overall naive algorithm (Algorithm 1) is divided into two chains that are run in parallel.

The auxiliary chain $h_{n}=\left({\tilde{\theta }}_{n},{\tilde{\varphi }}_{n}\right),n=0,1,2,\ldots ,$ uses stochastic approximation Monte Carlo (Liang et al. 2007) to estimate *p*(*Y|*φ) at a set of *m* pre-selected auxiliary parameter values ${\text{Φ}}_{0}=\left\{\varphi_{0}^{\left(1\right)},\ldots ,\varphi_{0}^{\left(m\right)}\right\}$ . Aswe detail in supplementary materials, the set Φ_0_ is chosen from a set of MCMC samples drawn from *p*(φ|*Z*), chosen using the Max–Min procedure (Liang et al. 2016) that repeatedly adds the sample that has the largest Euclidean distance to the hither to selected Φ_0_. This ensures that Φ_0_ covers the major part of the support of *p*(φ|*Z*). A reasonably large *m* ensures the distribution *p*(*θ*|*Y*, φ) overlaps each other for neighbouring $\varphi_{0}^{\left(a\right)}$ and $\varphi_{0}^{\left(b\right)}$ . The target density for $(\tilde{\theta },\tilde{\varphi })\in \text{Θ}\times {\text{Φ}}_{0}$ , which is proportional to *p*(*θ*|*Y*, φ) in (1) at the values Φ_0_, is

(3) $$p(\tilde{\theta },\tilde{\varphi })=\frac{1}{m}\sum_{i=1}^{m}\frac{p\left(Y|\tilde{\theta },\varphi_{0}^{\left(i\right)}\right)p(\tilde{\theta })}{p\left(Y|\varphi_{0}^{\left(i\right)}\right)}{𝟙}_{\left\{\tilde{\varphi }=\varphi_{0}^{\left(i\right)}\right\}}.$$

Given proposal distributions $q_{1}\left({\tilde{\theta }}^{\prime }|\tilde{\theta }\right)$ (e.g. symmetric random walk proposal) and $q_{2}\left({\tilde{\varphi }}^{\prime }|\tilde{\varphi }\right)$ (e.g. uniformly drawing ${\tilde{\varphi }}^{\prime }$ from a neighbouring set of $\tilde{\varphi })$ for $\tilde{\theta }$ and $\tilde{\varphi }$ individually, at each iteration *n*, proposals ${\tilde{\theta }}^{\prime }$ and ${\tilde{\varphi }}^{\prime }$ are drawn from amixture proposal distribution, with a fixed mixing probability *p*_mix_,

$$q\left({\tilde{\theta }}^{\prime },{\tilde{\varphi }}^{\prime }|{\tilde{\theta }}_{n-1},{\tilde{\varphi }}_{n-1}\right)=\{\begin{array}{l} p_{\text{mix}}q_{1}\left({\tilde{\theta }}^{\prime }|{\tilde{\theta }}_{n-1}\right),\;\text{for}\;{\tilde{\theta }}^{\prime }\neq {\tilde{\theta }}_{n-1}\\ \left(1-p_{\text{mix}}\right)q_{2}\left({\tilde{\varphi }}^{\prime }|{\tilde{\varphi }}_{n-1}\right),\;\text{for}\;{\tilde{\varphi }}^{\prime }\neq {\tilde{\varphi }}_{n-1}\\ 0\;\text{,otherwise} \end{array}$$

and accepted according to the Metropolis–Hastings acceptance probability with an iteration-specific target

$$p_{n}(\tilde{\theta },\tilde{\varphi })\propto \sum_{i=1}^{m}\frac{p\left(Y|\tilde{\theta },\varphi_{0}^{\left(i\right)}\right)p(\tilde{\theta })}{{\tilde{w}}_{n-1}^{\left(i\right)}}{𝟙}_{\left\{\tilde{\varphi }=\varphi_{0}^{\left(i\right)}\right\}},\tilde{\theta }\in \Theta ,\tilde{\varphi }\in {\text{Φ}}_{0}.$$

Here, ${\tilde{w}}_{n}^{\left(i\right)}$ is the estimate of $p\left(Y|\varphi_{0}^{\left(i\right)}\right)$ , *i* = 1,…, *m*,up to a constant, and ${\tilde{w}}_{n}=\left({\tilde{w}}_{n}^{\left(1\right)},\ldots ,{\tilde{w}}_{n}^{\left(m\right)}\right)$ is a vector of these estimates at each of the pre-selected auxiliary parameter values *Φ_0_*. We set ${\tilde{w}}_{0}^{\left(i\right)}=1$ , *i* = 1,…,*m* at the start. As described in Liang et al. (2007) and Liang et al. (2016), the estimates are updated by

(4) $$\log\left({\tilde{w}}_{n}^{\left(i\right)}\right)=\log\left({\tilde{w}}_{n-1}^{\left(i\right)}\right)+\xi_{n}\left(e_{n,i}-m^{-1}\right),i=1,\ldots ,m,$$

where *e_n_*,*i* = 1 if ${\tilde{\varphi }}_{n}=\varphi_{0}^{\left(i\right)}$ and *e_n_,i* = 0 otherwise, and *ζ_n_* = *n*_0_/ max(*n*_0_, *n*) decreases to 0 when *n* goes to infinity (the shrink magnitude *n*_0_ is a user-chosen fixed constant). Note that in this auxiliary chain, when the number of iterations is sufficiently large, we are drawing (*θ*, φ) from (3). Hence, by checking whether the empirical sampling frequency of each $\varphi_{0}^{\left(l\right)}\in {\text{Φ}}_{0}$ strongly deviates from *m* ^–1^, we can detect potential non-convergence of the auxiliary chain.

In the main Markov chain (*θ* *n*, φ*n*), *n* = 1, 2,…,we draw $\varphi^{\prime }$ from a proposal distribution $q\left(\varphi^{\prime }|\varphi \right)$ and then draw $\theta^{\prime }$ according to a random measure

(5) $$P_{n}^{*}\left(\theta \in ℬ|Y,\varphi^{\prime }\right)=\frac{\sum_{j=1}^{n}\sum_{i=1}^{m}{\tilde{w}}_{j-1}^{\left(i\right)}\frac{p\left(Y|{\tilde{\theta }}_{j},\varphi^{\prime }\right)}{p\left(Y|{\tilde{\theta }}_{j},\varphi_{0}^{\left(i\right)}\right)}{𝟙}_{\left\{{\tilde{\theta }}_{j}\in ℬ,\varphi_{0}^{\left(i\right)}={\tilde{\varphi }}_{j}\right\}}}{\sum_{j=1}^{n}\sum_{i=1}^{m}{\tilde{w}}_{j-1}^{\left(i\right)}\frac{p\left(Y|{\tilde{\theta }}_{j},\varphi^{\prime }\right)}{p\left(Y|{\tilde{\theta }}_{j},\varphi_{0}^{\left(i\right)}\right)}{𝟙}_{\left\{\varphi_{0}^{\left(i\right)}={\tilde{\varphi }}_{j}\right\}}},$$

where 𝓑 *⊂* *Θ* is any Borel set. Given a φ, the random measure (5) is formed via a dynamic importance sampling procedure proposed in Liang (2002) with intention to approximate the unknown distribution *p* (*θ* | *Y*, φ) (see supplementary material for a detailed explanation). For any Borel set 𝓑 *⊂* *Θ*,we have

$$\begin{array}{l} \begin{array}{l} \begin{array}{l} \frac{1}{n}\sum_{j=1}^{n}\sum_{i=1}^{m}{\tilde{w}}_{j-1}^{\left(i\right)}\frac{p\left(Y|{\tilde{\theta }}_{j},\varphi \right)p\left({\tilde{\theta }}_{j}\right)}{p\left(Y|{\tilde{\theta }}_{j},\varphi_{0}^{\left(i\right)}\right)p\left({\tilde{\theta }}_{j}\right)}{𝟙}_{\left\{{\tilde{\theta }}_{j}\in ℬ,\varphi_{0}^{\left(i\right)}={\tilde{\varphi }}_{j}\right\}}\\ \;\;\to \sum_{i=1}^{m}\int_{ℬ}mp\left(Y|\varphi_{0}^{\left(i\right)}\right)\frac{p(Y|\theta ,\varphi )p(\theta )}{p\left(Y|\theta ,\varphi_{0}^{\left(i\right)}\right)p(\theta )}\frac{1}{m}\frac{p\left(Y|\theta ,\varphi_{0}^{\left(i\right)}\right)p(\theta )}{p\left(Y|\varphi_{0}^{\left(i\right)}\right)}\text{d}\theta \end{array} \end{array}\\ \;\;=m\int_{ℬ}p(Y|\theta ,\varphi )p(\theta )\text{d}\theta , \end{array}$$

and similarly, the denominator of (5) converges to the *mp*(*Y* |φ). Hence, by Lemma 3.1 of Liang et al. (2016), since *Θ* × Φ is compact, for any Borel set 𝓑 ⊂ *Θ* and on any outcome ω of probability space Ω, we have:

(6) $$\underset{n\to \infty }{\lim}\underset{\varphi \in \Phi }{\sup}\left|P_{n}^{*}(\theta \in ℬ|Y,\varphi )-\int_{ℬ}p(\theta |Y,\varphi )\text{d}\theta \right|=0.$$

This implies that the distribution of {*θ* *n*}, drawn from (5), converges in distribution to *p*(*θ*|*Y*, φ), and this convergence occurs uniformly over Φ. Note that the probability measure $P_{n}^{*}\left(\theta \in ℬ|Y,\varphi^{\prime }\right)$ is adapted to filtration ${𝒢}_{n}=\sigma \left({\cup^{\text{​}}}_{j=1}^{n}\left({\tilde{\theta }}_{j},{\tilde{\varphi }}_{j},{\tilde{w}}_{j}\right)\right)$ on (Ω, 𝓕, ℙ) and has a Radon–Nikodym derivative with respect to a mixture of Dirac measures determined by ${\tilde{\Theta }}_{n}=\cup_{j=1}^{n}\left\{{\tilde{\theta }}_{j}\right\}$ (Gottardo and Raftery 2008), because it is the law of a discrete random variable defined on ${\tilde{\Theta }}_{n}$ . To achieve stronger convergence results, we will build a continuous probability distribution based on (5).

Algorithm 1 **Naive stochastic approximation cut algorithm**

Initialize at starting points $h_{0}=\left({\tilde{\theta }}_{0},{\tilde{\varphi }}_{0}\right),{\tilde{w}}_{0}\;\text{and}\;\left(\theta_{0},\varphi_{0}\right)$ ;

For *n* = 1,…,*N*;

(a) Auxiliary chain:
  (1) Draw a proposal $\left({\tilde{\theta }}^{\prime },{\tilde{\varphi }}^{\prime }\right)$ according to $q\left({\tilde{\theta }}^{\prime },{\tilde{\varphi }}^{\prime }|{\tilde{\theta }}_{n-1},{\tilde{\varphi }}_{n-1}\right)$ .
  (2) Accept the proposal, and set $\left({\tilde{\theta }}_{n},{\tilde{\varphi }}_{n}\right)=\left({\tilde{\theta }}^{\prime },{\tilde{\varphi }}^{\prime }\right)$ according to the iteration-specific acceptance probability.
  (3) Calculate ${\tilde{w}}_{n}^{\left(i\right)}$ according to (4), *i* = 1,…,*m*.
(b) Main chain:
  (1) Draw a proposal $\varphi^{\prime }$ according to $q\left(\varphi^{\prime }|\varphi_{n-1}\right)$ .
  (2) Set $\varphi_{n}=\varphi^{\prime }$ with probability:
$$\begin{array}{lll} \alpha \left(\varphi^{\prime }|\varphi_{n-1}\right) & \text{=} & \min\left\{1,\frac{p\left(\theta^{\prime }|Y,\varphi^{\prime }\right)p\left(\varphi^{\prime }|Z\right)q\left(\varphi_{n-1}|\varphi^{\prime }\right)p\left(\theta_{n-1}|Y,\varphi_{n-1}\right)}{p\left(\theta_{n-1}|Y,\varphi_{n-1}\right)p\left(\varphi_{n-1}|Z\right)q\left(\varphi^{\prime }|\varphi_{n-1}\right)p\left(\theta^{\prime }|Y,\varphi^{\prime }\right)}\right\}\\ \; & = & \min\left\{1,\frac{p\left(\varphi^{\prime }|Z\right)q\left(\varphi_{n-1}|\varphi^{\prime }\right)}{p\left(\varphi_{n-1}|Z\right)q\left(\varphi^{\prime }|\varphi_{n-1}\right)}\right\}. \end{array}$$
  (3) If $\varphi^{\prime }$ is accepted, draw $\theta^{\prime }$ according to $P_{n}^{*}\left(\theta^{\prime }|Y,\varphi^{\prime }\right)$ defined in (5) and set $\theta_{n}=\theta^{\prime }$ .
  (4) Otherwise if $\varphi^{\prime }$ is rejected, set (*θ*_*n*_, *φ*_*n*_) = (*θ*_*n*–_1, *φ*_*n*–_1).

End For;

### 2.2 Simple function approximation cut distribution

The convergence in distribution (6) presented in the naive stochastic approximation cut algorithm is not sufficiently strong to infer a law of large numbers or ergodicity of the drawn samples. We will show that these properties can be satisfied by targeting an approximation of the density function *p*(*θ*|*Y*,φ).

We adopt a density function approximation technique which uses a simple function as the basis. The use of a simple function to approximate a density function has been discussed previously (Fu and Wang 2002; Malefaki and Iliopoulos 2009), but here we use a different partition of the support of the function, determined by rounding to a user-specified number of decimal places. The general theory is presented in supplementary material.

Given the *d*-dimensional compact set *Θ* and user-specified number of decimal places κ, we partition *Θ* in terms of (partial) hypercubes *Θ*_*r*_ whose centres *θ*_*r*_ are the rounded elements of *Θ* to κ decimal places,

(7) $$\Theta_{r}=\Theta \cap \left\{\theta :{\left\|\theta -\theta_{r}\right\|}_{\infty }\leq 5\times 10^{-\kappa -1}\right\},r=1,\ldots ,R_{\kappa },$$

where *R*_κ_ is the total number of rounded elements. The boundary set ${\bar{\Theta }}_{K}$ , which has Lebesgue measure 0, is:

$${\bar{\Theta }}_{\kappa }=\Theta \cap (\overset{R_{\kappa }}{\underset{r=1}{\cup }}\left\{\theta :{\left\|\theta -\theta_{r}\right\|}_{\infty }=5\times 10^{-\kappa -1}\right\}).$$

Using this partition, we are able to build a simple function density that approximates *p*(*θ*|*Y*,φ):

$$p^{\left(\kappa \right)}(\theta |Y,\varphi )=\sum_{r=1}^{R_{K}}\frac{1}{\mu \left(\Theta_{r}\right)}\int_{\Theta_{r}}p\left(\theta^{\prime }|Y,\varphi \right)\text{d}\theta^{\prime }{𝟙}_{\left\{\theta \in \Theta_{r}\right\}},$$

and let *P* ^(κ)^ be the corresponding probability measure on *Θ*. The simple function approximation cut distribution is then formed by replacing the exact conditional distribution with this approximation

$$p_{\text{cut}\;}^{\left(\kappa \right)}(\theta ,\varphi )=p^{\left(\kappa \right)}(\theta |Y,\varphi )p(\varphi |Z).$$

Let $P_{\text{cut}}^{\left(\kappa \right)}$ be the corresponding probability measure on *Θ* *×*Φ.

Given the general theory presented in supplementary material, we have

(8) $$p^{\left(\kappa \right)}(\theta |Y,\varphi )\overset{\text{a}.\text{s}.\;}{\to }p(\theta |Y,\varphi ),\;\text{as}\;\kappa \to \infty .$$

The rate of convergence is tractable if we further assume density *p*(*θ* |*Y*, φ) is continuously differentiable.

**Corollary 1** *If density function p* (*θ* | *Y*, φ) *is continuously differentiable, there exists a set* ℰ *⊂* *Θ* *with* μ(ℰ) = μ(*Θ*) *such that the local convergence holds*:

$$\begin{array}{l} \left|p^{\left(\kappa \right)}(\theta |Y,\varphi )-p(\theta |Y,\varphi )\right|\\ \leq \left(\varepsilon (\theta ,\kappa )+\|\nabla p(\theta |Y,\varphi ){\|}_{2}\right)\frac{\sqrt{d}}{10^{\kappa }},\forall \theta \in ℰ, \end{array}$$

*where* ε(*θ*, κ) *→* 0 *as* κ *→∞*.

*In addition, the global convergence holds*:

$$\underset{\theta \in ℰ}{\sup}\left|p^{\left(\kappa \right)}(\theta |Y,\varphi )-p(\theta |Y,\varphi )\right|\leq \underset{\theta \in \Theta }{\sup}\|\nabla p(\theta |Y,\varphi ){\|}_{2}\frac{\sqrt{d}}{10^{\kappa }}.$$

**Proof** See the general theory in supplementary material.

### 2.3 Stochastic approximation cut algorithm

We now refine the naive stochastic approximation cut algorithm by replacing in the main chain the proposal distribution $P_{n}^{*}$ , which concentrates on the discrete set ${\tilde{\Theta }}_{n}$ , by a distribution, with support on the compact set *Θ*, that we will show converges almost surely to *P* ^(κ)^.

Let 𝒲*_n_* (φ) = (*W_n_* (*Θ* 1|*Y*, *φ*),…,*W_n_* (*Θ* *R*κ|*Y*, *φ*)) be a random weight process based on the probability of the original proposal distribution $P_{n}^{*}$ taking a value in each partition component *Θ* *r* as

(9) $$W_{n}\left(\Theta_{r}|Y,\varphi \right)=\frac{P_{n}^{*}\left(\theta \in \Theta_{r}|Y,\varphi \right)+{\left(nR_{\kappa }\right)}^{-1}}{1+n^{-1}},$$

where *r* = 1,…,*R*κ. Note that *W_n_* (*Θ* *r*|*Y*, *φ*) is adapted to the auxiliary filtration 𝒢_*n*_. By adding a (*nR*_κ_)^–1^, each *W_n_* (*Θ* *r|Y*,φ), *r* = 1, …, *R*κ, is strictly positive and yet this modification does not affect the limit since (*nR*_κ_)^–1^ *→* 0. That is, on any outcome ω of probability space Ω, we have

(10) $$\underset{n\to \infty }{\lim}\underset{\varphi \in \Phi ;1\leq r\leq R_{\kappa }}{\sup}\left|W_{n}\left(\Theta_{r}|Y,\varphi \right)-\int_{\Theta_{r}}p(\theta |Y,\varphi )\text{d}\theta \right|=0.$$

We now define the random measure process $P_{n}^{\left(\kappa \right)}$ that replaces $P_{n}^{*}$ used in the naive stochastic approximation cut algorithm. For any Borel set 𝓑,

(11) $$P_{n}^{\left(\kappa \right)}(\theta \in ℬ|Y,\varphi )=\int_{ℬ}\sum_{r=1}^{R_{\kappa }}\frac{1}{\mu \left(\Theta_{r}\right)}W_{n}\left(\Theta_{r}|Y,\varphi \right){𝟙}_{\left\{\theta \in \Theta_{r}\right\}}\text{d}\theta .$$

Clearly, $P_{n}^{\left(\kappa \right)}(\theta \in \text{Θ}|Y,\varphi )=1$ so $P_{n}^{\left(\kappa \right)}$ is a valid probability measure on *Θ*. Additionally, since 𝒲*n*(φ) is adapted to filtration 𝒢_*n*_, $P_{n}^{\left(\kappa \right)}$ is adapted to filtration 𝒢_*n*_. The Radon–Nikodym derivative of $P_{n}^{\left(\kappa \right)}$ with respect to the Lebesgue measure μ on *Θ* is

(12) $$p_{n}^{\left(\kappa \right)}(\theta |Y,\varphi )=\sum_{r=1}^{R_{K}}\frac{1}{\mu \left(\Theta_{r}\right)}W_{n}\left(\Theta_{r}|Y,\varphi \right){𝟙}_{\left\{\theta \in \Theta_{r}\right\}}.$$

This density is not continuous, but it is bounded on *Θ*. In addition, since *Θ* is the support of *p*(*θ* |*Y*, *φ*) and 𝒲*n*(*φ*) is strictly positive, the support of $P_{n}^{\left(\kappa \right)}$ is *Θ* for all *φ* *∈* Φ as well.

Using $P_{n}^{\left(\kappa \right)}$ as the proposal distribution has the advantage that $P_{n}^{\left(\kappa \right)}$ converges almost surely to *p* ^(κ)^, in contrast to the convergence in distribution for the naive algorithm in (6).

**Lemma 1** *Given* Assumption 1, *on any outcome* ω *of probability space Ω, we have*:

$$p_{n}^{\left(\kappa \right)}(\theta |Y,\varphi )\overset{\text{a}.\text{s}.}{\to }p^{\left(\kappa \right)}(\theta |Y,\varphi ),$$

*and this convergence is uniform over* $\left(\text{Θ}\setminus {\bar{\Theta }}_{K}\right)\times \text{Φ}$ .

Note that the convergence is to *p* ^(κ)^(*θ*|*Y*, *φ*) rather than *p*(*θ*|*Y*, *φ*), but we will show in Corollary 2 that this bias reduces geometrically as the precision parameterκ increases.

The complete stochastic approximation cut algorithm (SACut) is shown in Algorithm 2. The key idea is that we propose samples for *θ* from a density $p_{n}^{\left(\kappa \right)}(\theta |Y,\varphi )$ , which approximates *p*(*θ*|*Y*, *φ*) and from which we can draw samples, but we accept these proposals according to *p* ^(κ)^(*θ*|*Y*, *φ*), which then cancels. This results in the acceptance probability being determined only by the proposal distribution for *φ* the proposal distribution for *θ* is not involved. Indeed, the acceptance probability is the same as the partial Gibbs sampler that we will discuss in Sect. 3.1.1.

### 2.4 Parallelization and simplification of computation

The main computational bottleneck of the stochastic approximation cut algorithm is the updating and storage of the cumulative set of auxiliary variable values ${\tilde{\Theta }}_{n}=\cup_{j=1}^{n}\{{\tilde{\theta }}_{j}\}.$ Since we draw a new φ′ at each iteration, in order to calculate all possible probabilities defined by (5) and (9), the density $p(Y|\tilde{\theta },\phi^{\prime })$ must be calculated $\left|{\tilde{\Theta }}_{n}\right|$ times. This is equivalent to running $\left|{\tilde{\Theta }}_{n}\right|$ internal iterations at each step of external iteration for the existing approximate approaches proposed in Plummer (2015). Note that $\left|{\tilde{\Theta }}_{n}\right|$ is solely generated from the auxiliary chain so $\left|{\tilde{\Theta }}_{n}\right|$ is not affected by the precision parameter κ. If the calculation of this density is computationally expensive, the time to perform each update of the chain will become prohibitive when $p\left(Y|\tilde{\theta },\varphi^{\prime }\right)$ is large. However, the calculation of $\tilde{\theta }$ for different values of *θ* is embarrassingly parallel so can be evaluated in parallel whenever multiple computer cores are available, enabling a considerable speed up.

The speed of the computation can be further improved by reducing the size of ${\tilde{\Theta }}_{n}$ . Given the precision parameter κ, we

Algorithm 2 **Stochastic approximation cut (SACut) algorithm**

Initialize at starting points $h_{0}=({\tilde{\theta }}_{0},{\tilde{\varphi }}_{0}),{\tilde{w}}_{0}$ ;

For *n* = 1,*…,N* ;

(a) Auxiliary chain:
  (1) Draw a proposal $\left({\tilde{\theta }}^{\prime },{\tilde{\varphi }}^{\prime }\right)$ according to $q({\tilde{\theta }}^{\prime },{\tilde{\varphi }}^{\prime }{\tilde{\theta }}_{n-1},{\tilde{\varphi }}_{n-1}).$

  (2) Accept the proposal, and set $\left({\tilde{\theta }}_{n},{\tilde{\varphi }}_{n})=({\tilde{\theta }}^{\prime },{\tilde{\varphi }}^{\prime }\right)$ according to the iteration-specific acceptance probability.
  (3) Calculate ${\tilde{w}}_{n}^{\left(i\right)}$ according to (4), *i* = 1,*…, m*.
(b) Main chain:
  (1) Draw a proposal $\varphi^{\prime }$ according to $q(\varphi^{\prime }\varphi_{n-1})$ .
  (2) Set $\varphi_{n}=\varphi^{\prime }$ with probability:
$$\begin{array}{lll} \alpha \left(\varphi^{\prime }|\varphi_{n-1}\right) & \text{=} & \min\left\{1,\frac{p^{\left(\kappa \right)}\left(\theta^{\prime }|Y,\varphi^{\prime }\right)p\left(\varphi^{\prime }|Z\right)q\left(\varphi_{n-1}|\varphi^{\prime }\right)p^{\left(\kappa \right)}\left(\theta_{n-1}|Y,\varphi_{n-1}\right)}{p^{\left(\kappa \right)}\left(\theta_{n-1}|Y,\varphi_{n-1}\right)p\left(\varphi_{n-1}|Z\right)q\left(\varphi^{\prime }|\varphi_{n-1}\right)p^{\left(\kappa \right)}\left(\theta^{\prime }|Y,\varphi^{\prime }\right)}\right\}\\ \; & \text{=} & \min\left\{1,\frac{p\left(\varphi^{\prime }|Z\right)q\left(\varphi_{n-1}|\varphi^{\prime }\right)}{p\left(\varphi_{n-1}|Z\right)q\left(\varphi^{\prime }|\varphi_{n-1}\right)}\right\}. \end{array}$$
  (3) If $\varphi^{\prime }$ is accepted, calculate $W_{n}({\text{Θ}}_{r}Y,\varphi^{\prime })$ defined in (9), *r* = 1,…, *R_κ_*. Draw a proposal $\theta^{\prime }$ according to $p_{n}^{\left(\kappa \right)}(\theta^{\prime }Y,\varphi^{\prime })$ defined in (11) and set $\theta_{n}=\theta^{\prime }$ .
  (4) Otherwise if $\varphi^{\prime }$ is rejected, set (*θ*_*n*_, *φ*_*n*_) = (*θ*_*n*–_1, *φ*_*n*–_1).

End For;

round all elements from ${\tilde{\Theta }}_{n}$ to their κ decimal places and let ${\tilde{\Theta }}_{n}^{\left(\kappa \right)}$ be the set that contains these rounded elements. At each iteration, the number of calculations of density $p(Y\tilde{\theta },\varphi^{\prime })$ is equal to the number of *d*-orthotopes that auxiliary chain ${\left\{{\tilde{\theta }}_{j}\right\}}_{j=1}^{n}$ has visited up to iteration *n* and by (6) we know that the distribution of auxiliary samples of *θ̃* converges to the true distribution *p*(*θ*|*Y*, *φ*). Hence, the computational speed is mainly determined by the precision parameter κ and the targetdistribution *p* (*θ* | *Y*, *φ*). In particular, for any fixed κ and a sufficiently long auxiliary chain the computational cost is upper bounded by the case of uniform distribution since it equally distributes over the space *Θ*.

**Theorem 1** *Given an arbitrary d-dimensional compact parameter space* *Θ* *and a precision parameter* κ *and suppose that the auxiliary chain has converged before we start collecting auxiliary variable *θ̄*, for any fixed number of iteration n. Then, the expected number of d-orthotopes visited* $E\left(\left|{\tilde{\Theta }}_{n}^{\left(\kappa \right)}\right|\right)$ *is maximized when the target distribution is uniform distribution*.

**Proof** See supplementary material (Online Resource 1).

For example, given a *d*-dimensional parameter space *Θ* = [0*–*5*×*10^*–*κ*–*1^, 1*+*5*×*10^*–*κ*–*1^]*^d^* and its partition *Θ*_*r*_, *r* = 1, …, 11^dκ^, we consider the uniform distribution as the target distribution. Assuming the auxiliary chain has converged, the expectation of $\left|{\tilde{\Theta }}_{n}^{\left(\kappa \right)}\right|$ is

$$E\left(\left|{\tilde{\Theta }}_{n}^{\left(\kappa \right)}\right|\right)=11^{\text{d}\kappa }-\frac{{\left(11^{\text{d}\kappa }-1\right)}^{n}}{11^{\text{d}\kappa (n-1)}}.$$

**Fig. 3:**
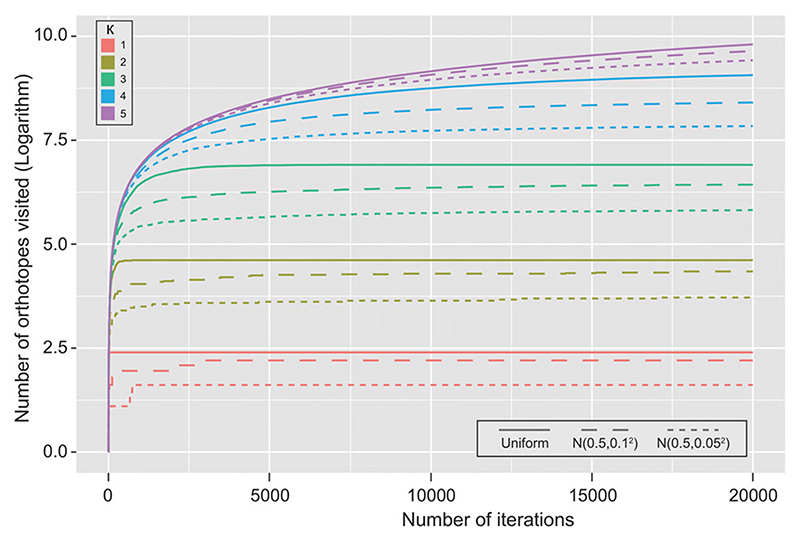
Relationship between the number of orthotopes visited and the number of iterations when precision parameter κ *=* 1, 2, 3, 4, 5. Separate Monte Carlo simulations were conducted for uniform distribution and truncated normal distribution with standard deviation 0.1 and 0.05

In the case of *d* = 1, Fig. 3 compares the number of orthotopes visited between the uniform distribution and truncated normal distribution when the standard deviation is 0.1 and 0.05. It shows that larger precision parameter κ means more evaluations of $p(Y|\tilde{\theta },\varphi^{\prime })$ are required. Hence, a wise choice of a small *κ* can significantly reduce computation time.

While small *κ* means a loss of precision since local variation so f original target distribution are smoothed by rounding the value of its samples, in most applied settings only a small number of significant figures are meaningful, and so the ability to trade-off the precision and computational speed is appealing. Comparing short preliminary run of chains for different candidates of *κ* may be useful when a suitable choice of *κ* is unclear. We will discuss this in Sect 4.1.

## 3 Convergence properties

In this section, we study the convergence properties of samples drawn by the stochastic approximation cut algorithm. We establish a weak law of large numbers with respect to the simple function approximation cut distribution $P_{\text{cut}}^{\left(\kappa \right)}$ , under some regularity conditions, by proving that the conditions required by Theorem 3.2 in Liang et al. (2016) are satisfied. We then prove that the bias with respect to *p*_cut_ can be reduced geometrically by increasing the precision parameter *κ*. To aid exposition of the convergence properties, it is necessary to first introduce two simpler but infeasible alternative algorithms. Then, we prove the convergence of the algorithm.

The framework of our proofs follow Liang et al. (2016). However, adjustments are made for two key differences. Firstly, the parameter of interest here has two components,instead of just one, and we require completely different proposal distributions to those in Liang et al. (2016): the proposal distribution of *θ* involves an auxiliary chain and simple function approximation, and the proposal distribution of *φ* is a standard MCMC algorithm. Secondly, the parameter drawn by (12) here is retained, rather than being discarded as in Liang et al. (2016). This means the distributions involved here are different and more complicated.

### 3.1 Infeasible alternative algorithms

**Definition 1** Given a signed measure 𝑀 defined on a set *E*, and a Borel set 𝓑 ⊂ *E*, define the total variation norm of 𝑀 as

$$\|ℳ(\cdot ){\|}_{\text{TV}}=\underset{ℬ\subset E}{\sup}|ℳ(ℬ)|.$$

#### 3.1.1 A partial Gibbs sampler

The most straightforward algorithm that draws samples from $p_{\text{cut}}^{\left(\kappa \right)}(\theta ,\varphi )$ is a standard partial Gibbs sampler, which draws proposals $\theta^{\prime }$ from $p^{\left(\kappa \right)}(\theta^{\prime }|Y,\varphi^{\prime }),$ , given a $\varphi^{\prime }$ drawn from a proposal distribution $q(\varphi^{\prime }|\varphi_{n-1}).$ . The transition kernel is

$$\begin{array}{l} {\text{u}}^{\left(1\right)}\left(\left(\theta_{n},\varphi_{n}\right)|\left(\theta_{n-1},\varphi_{n-1}\right)\right)\\ \;=\alpha \left(\varphi_{n}|\varphi_{n-1}\right)p^{\left(\kappa \right)}\left(\theta_{n}|Y,\varphi_{n}\right)q\left(\varphi_{n}|\varphi_{n-1}\right)\\ \;+\left(1-\int_{\Theta \times \Phi }\alpha \left(\varphi |\varphi_{n-1}\right)p^{\left(\kappa \right)}(\theta |Y,\varphi )q\left(\varphi |\varphi_{n-1}\right)\text{d}\theta \text{d}\varphi \right)\\ \;\delta \left(\left(\theta_{n},\varphi_{n}\right)-\left(\theta_{n-1},\varphi_{n-1}\right)\right)\\ \;=\alpha \left(\varphi_{n}|\varphi_{n-1}\right)p^{\left(\kappa \right)}\left(\theta_{n}|Y,\varphi_{n}\right)q\left(\varphi_{n}|\varphi_{n-1}\right)\\ \;+\left(1-\int_{\Phi }\alpha \left(\varphi |\varphi_{n-1}\right)q\left(\varphi |\varphi_{n-1}\right)\text{d}\varphi \right)\\ \;\delta \left(\left(\theta_{n},\varphi_{n}\right)-\left(\theta_{n-1},\varphi_{n-1}\right)\right), \end{array}$$

where δ is the multivariate Dirac delta function and

$$\alpha \left(\varphi_{n}|\varphi_{n-1}\right)=\min\left\{1,\frac{p\left(\varphi_{n}|Z\right)q\left(\varphi_{n-1}|\varphi_{n}\right)}{p\left(\varphi_{n-1}|Z\right)q\left(\varphi_{n}|\varphi_{n-1}\right)}\right\}.$$

This transition kernel is Markovian and admits $P_{\text{cut}}^{\left(\kappa \right)}$ as its stationary distribution, provided a proper proposal distribution *q*(*φ_n_* |*φ_n_*–1) is used. We write **U** ^(1)^ for the corresponding probability measure.

Let **u** ^(*s*)^ denote the s-step transition kernel and write **U** ^(*s*)^ for the corresponding probability measure. By Meyn et al. (2009), we have ergodicity on *Θ* × Φ,

$$\underset{s\to \infty }{\lim}{\left\|{\text{U}}^{\left(s\right)}(\cdot )-P_{\text{cut}\;}^{\left(\kappa \right)}(\cdot )\right\|}_{\text{TV}}=0,$$

and for any bounded function *f* defined on *Θ* × *Φ*, we have a strong law of large numbers

$$\frac{1}{N}\sum_{n=1}^{N}f\left(\theta_{n},\varphi_{n}\right)\overset{\text{a}.\text{s}.}{\to }\int_{\Theta \times \Phi }f(\theta ,\varphi )P_{\text{cut}\;}^{\left(\kappa \right)}(\text{d}\theta ,\text{d}\varphi ).$$

Note, however, that this algorithm is infeasible because *p* ^(κ)^(*θ*|*Y*, *φ*) is intractable, since *p*(*θ*|*Y*, *φ*) is intractable, and so we cannot directly draw proposals for *θ*.

#### 3.1.2 An adaptive Metropolis–Hastings sampler

An adaptive Metropolis–Hastings sampler can be built by replacing *p* ^(κ)^ in the calculation of acceptance probability of the stochastic approximation cut algorithm by its approximation $p_{n}^{\left(\kappa \right)}$ , which is the exact proposal distribution for *θ* at the *n*th step. The acceptance probability is determined by both *θ* and *φ*,

$$\begin{array}{l} \alpha_{n}\left(\left(\theta^{\prime },\varphi^{\prime }\right)|\left(\theta_{n-1},\varphi_{n-1}\right)\right)\\ \;=\min\{1,\frac{p^{\left(\kappa \right)}\left(\theta^{\prime }|Y,\varphi^{\prime }\right)p\left(\varphi^{\prime }|Z\right)q\left(\varphi_{n-1}|\varphi^{\prime }\right)p_{n}^{\left(K\right)}\left(\theta_{n-1}|Y,\varphi_{n-1}\right)}{p^{\left(\kappa \right)}\left(\theta_{n-1}|Y,\varphi_{n-1}\right)p\left(\varphi_{n-1}|Z\right)q\left(\varphi^{\prime }|\varphi_{n-1}\right)p_{n}^{\left(K\right)}\left(\theta^{\prime }|Y,\varphi^{\prime }\right)}\}, \end{array}$$

and we can write the transition kernel,

$$\begin{array}{l} {\text{v}}_{n}^{\left(1\right)}\left(\left(\theta_{n},\varphi_{n}\right)|\left(\theta_{n-1},\varphi_{n-1}\right),{𝒢}_{n}\right)\\ \;=\alpha_{n}\left(\left(\theta_{n},\varphi_{n}\right)|\left(\theta_{n-1},\varphi_{n-1}\right)\right)p_{n}^{\left(\kappa \right)}\left(\theta_{n}|Y,\varphi_{n}\right)q\left(\varphi_{n}|\varphi_{n-1}\right)\\ \;+(1-\int_{\Theta \times \Phi }\alpha_{n}\left((\theta ,\varphi )|\left(\theta_{n-1},\varphi_{n-1}\right)\right)p_{n}^{\left(\kappa \right)}(\theta |Y,\varphi )\\ q\left(\varphi |\varphi_{n-1}\right)\text{d}\theta \text{d}\varphi )\delta \left(\left(\theta_{n},\varphi_{n}\right)-\left(\theta_{n-1},\varphi_{n-1}\right)\right), \end{array}$$

where δ is the multivariate Dirac delta function. Conditional on the filtration 𝒢_*n*_, ${\text{v}}_{n}^{\left(1\right)}$ is Markovian. We write ${\text{V}}_{n}^{\left(1\right)}$ for the corresponding probability measure. Note that this sampler is not a standard Metropolis–Hastings algorithm since the transition kernel is not constant. Instead, it is an *external* adaptive MCMC algorithm (Atchadé et al. 2011).

Given information up to 𝒢_*n*_, if we stop updating auxiliary process, then $P_{n}^{\left(\kappa \right)}$ is fixed and not random, and this sampler reduces to a standard Metropolis–Hastings sampler. The transition kernel ${\text{V}}_{n}^{\left(1\right)}$ admits $p_{\text{cut}\;}^{\left(\kappa \right)}$ as its stationary distribution provided a proper proposal distribution is used. That is, define

$$\begin{array}{l} {\text{v}}_{n}^{\left(s\right)}=\int_{\Theta^{s-1}\times \Phi^{s-1}}\prod_{k=1}^{s}{\text{v}}_{n}^{\left(1\right)}\left(\left(\theta_{k},\varphi_{k}\right)|\left(\theta_{k-1},\varphi_{k-1}\right),{𝒢}_{n}\right)\\ \;\text{d}\theta_{1:s-1}\;\text{d}\varphi_{1:s-1}, \end{array}$$

and ${\text{V}}_{n}^{\left(s\right)}$ as the corresponding probability measure. Then, on *Θ* × Φ we have

$$\underset{s\to \infty }{\lim}{\left\|{\text{V}}_{n}^{\left(s\right)}(\cdot )-P_{\text{cut}}^{\left(\kappa \right)}(\cdot )\right\|}_{\text{TV}}=0.$$

Note, however, that this algorithm is also infeasible because, while we can draw proposals for *θ*, since $p_{n}^{\left(\kappa \right)}$ is known up to 𝒢*_n_*, *p* ^(κ)^(*θ*|*Y*, φ) remains intractable so we cannot calculate the acceptance probability.

### 3.2 Convergence of the stochastic approximation cut algorithm

The infeasibility of the partial Gibbs sampler and the adaptive Metropolis–Hastings sampler motivates the development of the stochastic approximation cut algorithm, which replaces the proposal distribution $p_{n}^{\left(\kappa \right)}$ by its target *p* ^(κ)^ in the accept–reject step of the adaptive Metropolis–Hastings sampler. This leads to the same acceptance probability as is used by the partial Gibbs sampler, so the proposed algorithm can be viewed as combining the advantages of both the partial Gibbs sampler and the adaptive Metropolis–Hastings sampler. The transition kernel of the stochastic approximation cut algorithm is

$$\begin{array}{l} {\text{t}}_{n}^{\left(1\right)}\left(\left(\theta_{n},\varphi_{n}\right)|\left(\theta_{n-1},\varphi_{n-1}\right),{𝒢}_{n}\right)\\ \;=\alpha \left(\varphi_{n}|\varphi_{n-1}\right)p_{n}^{\left(\kappa \right)}\left(\theta_{n}|Y,\varphi_{n}\right)q\left(\varphi_{n}|\varphi_{n-1}\right)\\ \;+\left(1-\int_{\Theta \times \Phi }\alpha \left(\varphi |\varphi_{n-1}\right)p_{n}^{\left(\kappa \right)}(\theta |Y,\varphi )q\left(\varphi |\varphi_{n-1}\right)\text{d}\theta \text{d}\varphi \right)\\ \;\delta \left(\left(\theta_{n},\varphi_{n}\right)-\left(\theta_{n-1},\varphi_{n-1}\right)\right)\\ \;=\alpha \left(\varphi_{n}|\varphi_{n-1}\right)p_{n}^{\left(\kappa \right)}\left(\theta_{n}|Y,\varphi_{n}\right)q\left(\varphi_{n}|\varphi_{n-1}\right)\\ \;+\left(1-\int_{\Phi }\alpha \left(\varphi |\varphi_{n-1}\right)q\left(\varphi |\varphi_{n-1}\right)\text{d}\varphi \right)\\ \;\delta \left(\left(\theta_{n},\varphi_{n}\right)-\left(\theta_{n-1},\varphi_{n-1}\right)\right), \end{array}$$

where δ is the multivariate Dirac delta function. Conditionally to 𝒢_*n*_, the transition kernel ${\text{t}}_{n}^{\left(1\right)}$ is Markovian. We write ${\text{T}}_{n}^{\left(1\right)}$ for the corresponding probability measure. Given information up to 𝒢_*n*_ and stopping updating the auxiliary process, $P_{n}^{\left(\kappa \right)}$ is fixed and not random, and we define the *s*-step transition kernel as

$$\begin{array}{l} {\text{t}}_{n}^{\left(s\right)}=\int_{\Theta^{s-1}\times \Phi^{s-1}}\prod_{k=1}^{s}{\text{t}}_{n}^{\left(1\right)}\left(\left(\theta_{k},\varphi_{k}\right)|\left(\theta_{k-1},\varphi_{k-1}\right),{𝒢}_{n}\right)\\ \;\;\;\;\;\;\;\;\;\;\;\;\text{d}\theta_{1:s-1}\;\text{d}\varphi_{1:s-1}, \end{array}$$

and write ${\text{T}}_{n}^{\left(s\right)}$ for the corresponding probability measure.

We now present several lemmas required to prove a weak law of large numbers for this algorithm (proofs in supplementary material (Online Resource 1)), appropriately modifying the reasoning of Meyn and Tweedie (1994), Roberts and Tweedie (1996) and Liang et al. (2016) for this setting.

**Assumption 2** The posterior density *p*(*φ*|*Z*) is continuous on Φ and the proposal distribution $q\left(\varphi^{\prime }|\varphi \right)$ is continuous with respect to $\left(\varphi^{\prime },\varphi \right)$ on Φ × Φ.

**Lemma 2** (Diminishing adaptation) *Given* Assumptions 1 *and* 2, *then*

$$\underset{n\to \infty }{\lim}\underset{\theta \in \Theta ,\varphi \in \Phi }{\sup}{\left\|T_{n+1}^{\left(1\right)}\left(\cdot |(\theta ,\varphi ),{𝒢}_{n+1}\right)-T_{n}^{\left(1\right)}\left(\cdot |(\theta ,\varphi ),{𝒢}_{n}\right)\right\|}_{\text{TV}}=0.$$

Before presenting the next lemma, we introduce the concept of *local positivity*.

**Definition 2** A proposal distribution $q\left(\psi^{\prime }\psi \right)$ satisfies local positivity if there exists δ > 0 and ε > 0 such thatfor every $\psi \in \text{Ψ},\left|\psi^{\prime }-\psi \right|\leq \delta$ implies that $q\left(\psi^{\prime }\psi \right)>\varepsilon$ .

**Lemma 3** *Given* Assumption 1, *the proposal distributions with densities $p_{n}^{\left(\kappa \right)}:\text{Θ}\to \mathbb{R}$ *and p* ^(κ)^ : *Θ* → ℝ *are both uniformly lower bounded away from* 0 *and satisfy local positivity uniformly for all values* *φ** ∈ Φ.

**Lemma 4** (Stationarity) *Given* Assumptions 1 and 2, *and the filtration* 𝒢*_n_* (*i*.*e*. $P_{n}^{\left(\kappa \right)}$ *is not random*), *then if the transition kernel measures **U*** ^(1)^ *and* $V_{n}^{\left(1\right)}$ *both admit an irreducible and aperiodic Markov chain, then the transition kernel measure* $T_{n}^{\left(1\right)}$ *admits an irreducible and aperiodic chain. Moreover, if the proposal distribution* $q\left(\varphi^{\prime }\varphi \right)$ *satisfies local positivity, then there exists a probability measure ⊓_n_ on* *Θ* × Φ *such that for any* (*θ*_0_, *φ*_0_) ∈ *Θ* × Φ,

$$\underset{s\to \infty }{\lim}{\left\|T_{n}^{\left(s\right)}(\cdot )-\Pi_{n}(\cdot )\right\|}_{\text{TV}}=0,$$

*and this convergence is uniform over* *Θ* × Φ.

**Lemma 5** (Asymptotic Simultaneous Uniform Ergodicity) *Given* Assumptions 1 *and* 2 *and the assumptions in Lemma 4, for any initial value* (*θ*_0_, *φ*_0_) ∈ *Θ* × Φ, *and any*ε > 0 *and e* > 0, *there exist constants S*(ε) > 0 *and N*(ε) > 0 *such that*

$$\mathbb{P}\left(\left\{P_{n}^{\left(\kappa \right)}:{\left\|T_{n}^{\left(s\right)}(\cdot )-P_{\text{cut}\;}^{\left(\kappa \right)}(\cdot )\right\|}_{\text{TV}}\leq \varepsilon \right\}\right)>1-e,$$

*for alls* > *S*(ε) *and n* > *N*(ε).

Lemma 2 leads to condition (c) (diminishing adaptation), Lemma 4 leads to condition (a) (stationarity) and Lemma 5 leads to condition (b) (asymptotic simultaneous uniform ergodicity) in Theorem 3.2 of Liang etal.(2016). Hence,we have the following weak law of large numbers.

**Theorem 2** (WLLN) *Suppose that the conditions of Lemma 5 hold. Let *f* be any measurable bounded function on* *Θ*×Φ*. Then, for samples* (*θ_n_*, *φ_n_*), *n* = 1, 2, … *drawn using the stochastic approximation cut algorithm, we have that*

$$\frac{1}{N}\sum_{n=1}^{N}f\left(\theta_{n},\varphi_{n}\right)\to \int_{\Theta \times \Phi }f(\theta ,\varphi )P_{\text{cut}}^{\left(\kappa \right)}(\text{d}\theta ,\text{d}\varphi ),$$

*in probability*.

**Proof** This follows by Theorem 3.2 in Liang et al. (2016).

Given further conditions and combining Corollary 1 with Theorem 2, we have the following corollary.

**Corollary 2** *Given the conditions in Corollary 1 hold for the cut distribution p*cut *and conditions in Theorem 2 hold. Then, given a measurable and bounded function f* : *Θ* ×Φ → R, *there exists, for any* ε > 0 *and e* > 0, *a precision parameter* κ *and iteration number N, such that for samples* (*θ* *_n_*, *φ_n_*), *n* = 1, 2, … *drawn using the stochastic approximation cut algorithm, we have that*

$$\mathbb{P}\left(\left|\frac{1}{N}\sum_{n=1}^{N}f\left(\theta_{n},\varphi_{n}\right)-\int_{\Theta \times \Phi }f(\theta ,\varphi )P_{\text{cut}}(\text{d}\theta ,\text{d}\varphi )\right|\leq \varepsilon \right)>1-e.$$

*More specifically, the bias*

$$\left|\int_{\Theta \times \Phi }f(\theta ,\varphi )P_{\text{cut}}(\text{d}\theta ,\text{d}\varphi )-\int_{\Theta \times \Phi }f(\theta ,\varphi )P_{\text{cut}}^{\left(\kappa \right)}(\text{d}\theta ,\text{d}\varphi )\right|$$

*can be controlled by*

$$\underset{\theta \in \Theta ,\varphi \in \Phi }{\sup}{\left\|\nabla_{\theta }p(\theta |Y,\varphi )\right\|}_{2}\frac{\sqrt{d}}{10^{\kappa }}\left(\int_{\Theta \times \Phi }f(\theta ,\varphi )p(\varphi |Z)\text{d}\theta \text{d}\varphi \right).$$

Corollary 2 shows that, although the convergence established by Theorem 2 is biased with respect to the true cut distribution *p*_cut_, the bias can be geometrically reduced by selecting a large precision parameter κ.

## 4 Illustrative examples

We demonstrate the proposed algorithm in this section. First, we use as imulation example to introduce a simple method for choosing the precision parameter κ and demonstrate that the proposed algorithm can eliminate the feedback from a suspect module. We then examine a simulated case designed to highlight when existing algorithms will perform poorly. We finally apply our algorithm to an epidemiological example and compare results with existing studies. The R package *SACut* and code to replicate these examples can be downloaded from GitHub.^1^

### 4.1 Simulated random effects example

In this example, we discuss a simple method for selecting the precision parameter κ and show that the proposed algorithm can effectively cut the feedback from a suspect module.

We consider a simple normal–normal random effect example previously discussed by Liu et al. (2009), with groups *i* = 1,…,100 = *N*, observations $Y_{ij}∼\text{N}\left(\beta_{i},\varphi_{i}^{2}\right),j=1,\ldots ,20$ in each group, and random effects distribution β*_i_* ∼ N(0,*θ* ^2^). Our aim is to estimate the random effects standard deviation *θ* and the residual standard deviation *φ* = (*φ*_1_,…,φ*_N_*). By sufficiency, the likelihood can be equivalently represented in terms of the group-specific means ${\bar{Y}}_{i}=\frac{1}{20}\sum_{j=1}^{20}Y_{ij}$ and the sum of squared deviations $s_{i}^{2}=\sum_{j=1}^{20}{\left(Y_{ij}-{\bar{Y}}_{i}\right)}^{2}$ as

$$\begin{array}{l} {\bar{Y}}_{i}\sim \text{N}\left(\beta_{i},\frac{\varphi_{i}^{2}}{20}\right),\\ s_{i}^{2}\sim \;\text{Gamma}\;\left(\frac{20-1}{2},\frac{1}{2\varphi_{i}^{2}}\right). \end{array}$$

Given the sufficient statistics $\bar{Y}=\left({\bar{Y}}_{1},\ldots ,{\bar{Y}}_{N}\right)$ and $s^{2}=\left(s_{1}^{2},\ldots ,s_{N}^{2}\right)$ , the model consists of two modules: module 1 involving (*s* ^2^, *φ*) and module 2 involving $\left(\bar{Y},\beta ,\varphi \right)$ , where *β* = (*β*_1_,…,*β_N_*).

We consider the situation when an outlier group is observed, meaning that module 2 is misspecified, and compare the standard Bayesian posterior distribution with the cut distribution. Specifically, we simulate data from the model with *θ* ^2^ = 2, and $\varphi_{i}^{2}$ drawn from a Unif(0.5, 1.5) distribution $\left(\varphi_{1}^{2}=1.60\right)$ , but we artificially set β_1_ = 10, making the first group an outlier and thus our normal assumption for the random effects misspecified. Given priors $p\left(\varphi_{i}^{2}\right)\propto {\left(\varphi_{i}^{2}\right)}^{-1}$ and $p\left(\theta^{2}\varphi^{2}\right)\propto {\left(\theta^{2}+{\bar{\varphi }}^{-2}/20\right)}^{-1}$ , Liu et al. (2009) showed the standard Bayesian marginal posterior distribution for the parameters of interest is:

$$\begin{array}{l} p\left(\theta ,\varphi |\bar{Y},s^{2}\right)=p(\theta |\bar{Y},\varphi )p\left(\varphi |\bar{Y},s^{2}\right)\\ \;\propto \frac{1}{\theta^{2}+{\bar{\varphi }}^{2}/20}\prod_{i=1}^{100}{\left(\varphi_{i}^{2}\right)}^{-\frac{21}{2}}\exp\left(-\frac{s_{i}^{2}}{2\varphi_{i}^{2}}\right)\\ \;\frac{1}{{\left(\theta^{2}+\varphi_{i}^{2}/20\right)}^{1/2}}\exp\left(-\frac{{\bar{Y}}_{i}^{2}}{2\left(\theta^{2}+\varphi_{i}^{2}/20\right)}\right). \end{array}$$

Since we are confident about our assumption of normality of *Y_ij_* but not confident about our distributional assumption for the random effects β*i*, following Liu et al. (2009), we consider the cut distribution in which we remove the influence of $\bar{Y}$ on *φ*, so that possible misspecification of the first module does not affect *φ*:

$$p_{\text{cut}\;}(\theta ,\varphi ):=p(\theta |\bar{Y},\varphi )p\left(\varphi |s^{2}\right),$$

where

$$p\left(\varphi |s^{2}\right)\propto \prod_{i=1}^{100}\varphi_{i}^{-21}\exp\left(-\frac{s_{i}^{2}}{2\varphi_{i}^{2}}\right).$$

To apply the proposed algorithm we first construct the auxiliary parameter set for the parameter *φ* by selecting 70 samples selected from posterior samples of *p*(*φ*|*s* ^2^) by the Max-Min procedure (Liang et al. 2016). We set the shrink magnitude *n*_0_ = 1000 and run only the auxiliary chain for 10^4^ iterations before starting to store the auxiliary variable *h_n_*, as suggested by Liang et al. (2016).

The precision parameter κ should be chosen large enough to obtain accurate results, while being small enough that computation is not prohibitively slow. To illustrate this, we compare results with κ = 10, which we regard as the gold standard, to results with κ = 1, 2, 3, 4. Different values of κ affect the sampling of *θ* only via (11), so we compare samples drawn from $p_{n}^{\left(\kappa \right)}(\theta |\bar{Y},\varphi )$ , averaged over the marginal cut distribution of *φ*:

(13) $$p_{n}^{\left(\kappa \right)}\left(\theta |\bar{Y},s^{2}\right):=\int p_{n}^{\left(\kappa \right)}(\theta |\bar{Y},\varphi )p_{\text{cut}}(\varphi )\text{d}\varphi ,$$

where the marginal cut distribution *p*_cut_(*φ*) is

$$p_{\text{cut}}(\varphi ):=\int p_{\text{cut}}(\theta ,\varphi )\text{d}\theta =p\left(\varphi |s^{2}\right)\propto p\left(s^{2}|\varphi \right)p(\varphi ).$$

**Fig. 4:**
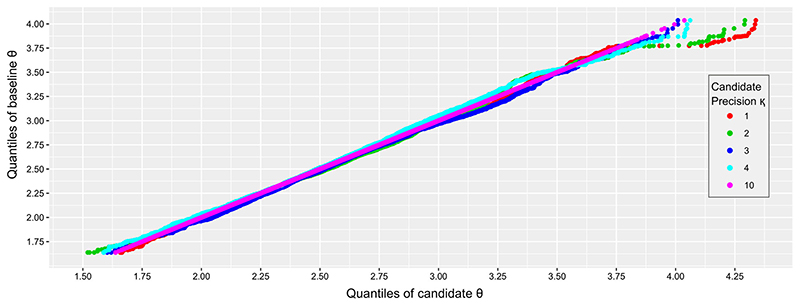
Quantile–quantile plot for *θ* drawn from (13) with precision parameter κ = 1,2,3,4,10. The*x*-axis of the quantile–quantile plot is the quantile of samples under different κ, and the y-axis is the quantile of samples under the gold standard κ = 10

We draw 10^5^ samples from (13) for each value of κ, after running the proposed algorithm with few iterations (10^4^) as a preliminary trial. Figure 4 shows the quantile–quantile plot for 5 choices for κ. The fit appears good for all choices of κ, except in the tails, where κ = 3 and κ = 4 provide a closer match to the gold standard. Thus, we choose κ = 3 as it gives a sufficiently accurate approximation.

**Fig. 5:**
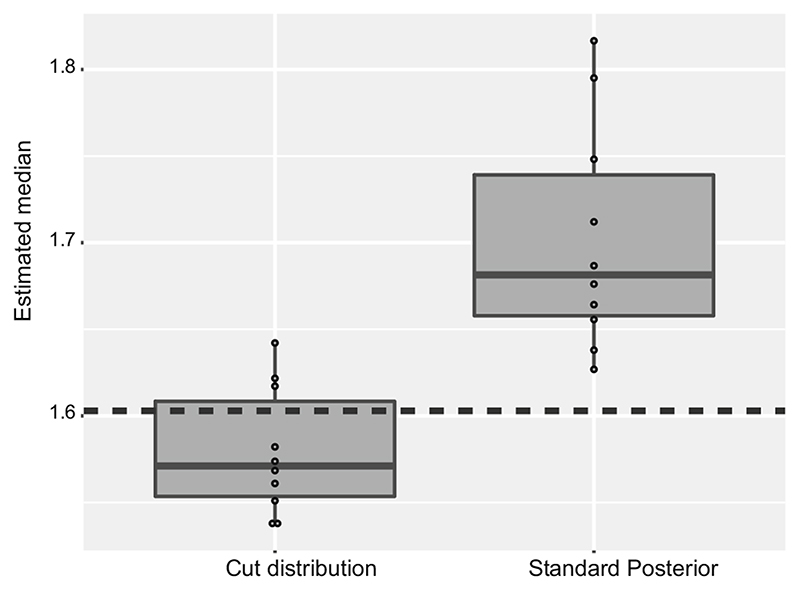
Box plot ofmedian estimates for $\varphi_{1}^{2}$ from each of ten independent runs, under the cut distribution and the standard Bayesian posterior. The dashed line indicates the true value of $\varphi_{1}^{2}$

We apply both the standard Bayesian approach and the stochastic approximation cut algorithm (κ = 3), each with ten independent chains. All chains were run for 10^5^ iterations, and we retain only every 100th value, after discarding the first 10% of the samples, and we summarize the results by the mean and credible interval (CrI). Pooling the ten chains for the cut distribution gave estimates of *θ* ^2^ = 2.54 (95% CrI 1.93–3.44)and $\varphi_{1}^{2}=1.58(95\text{\%CrI}0.88-3.18)$ , where as the standard Bayesian approach gave estimates of *θ* ^2^ = 2.53 (95% CrI 1.93–3.44) and $\varphi_{1}^{2}=1.69(95\text{\%CrI}0.91-3.76)$ . Figure 5 presents the medians for the parameter of interest $\varphi_{1}^{2}$ under each of the ten independent runs for the cut distribution and the standard Bayesian posterior. Recalling the true value for $\varphi_{1}^{2}=1.60$ , it is clear that when using the stochastic approximation cut algorithm the medians locate around its true value rather than deviating systematically towards one side. This indicates the proposed algorithm has successfully prevented the outlying observation from influencing the estimation of $\varphi_{1}^{2}$ .

### 4.2 Simulated strong dependence between *θ* and *ϕ*

In this section, we apply our algorithm in a simulated setting that illustrates when nested MCMC (Plummer 2015) can perform poorly. Consider the case when the distribution of *θ* is highly dependent on *φ*. In this case, if the distance between successive values $\varphi^{\prime }$ and *φ* is large in the external MCMC chain, the weight function may not be close to 1 and so the internal chain will typically require more iterations to reach convergence. This will be particularly problematic if the mixing time for the proposal distribution is large.

To simulate this scenario, we consider a linear regression for outcomes *Y_i_*, *i* = 1,…,50, in which the coefficient vector *θ* = (*θ*_1_,…,*θ* *_d_*) is closely related to the coefficient *φ* for covariate *X_i_* = (*X* *θ*,*i*, *X*φ,*i*). To assess the performance under small and moderate dimension of *θ*, we consider *d* = 1 and 20 in this illustration. In addition to observations of the outcome *Y_i_* and the covariate *X_i_*, we assume we have separate observations *Z_j_*, *j* = 1, …, 100 related to the coefficient *φ*.

(14) $$\begin{array}{l} Y_{i}\sim \text{N}\left(\theta^{⊤}X_{\theta ,i}+\varphi X_{\varphi ,i},3\right),i=1,\ldots ,50;\\ Z_{j}\sim \text{N}(\varphi ,1),j=1,\ldots ,100. \end{array}$$

Suppose that we wish to estimate *φ* solely on the basis of *Z* = (*Z*_1_,…, *Z*_100_), and so we cut the feedback from *Y* = (*Y*_1_,…,*Y*_50_) to *φ*.

We generate *Y* and *Z* according to (14), with *φ* = 1 and *θ* *p* = sin(*p*), *p* = 1,…,*d*, and compare the results of stochastic approximation cut (SACut) algorithm, naive SACut and nested MCMC with internal chain length *n*_int_ =1, 10, 200, 500, 1000, 1500 and 2000. Notably, nested MCMC with *n*_int_ = 1 is the WinBUGS algorithm. The proposal distribution for each element of *φ* is a normal distribution, centred at the previous value and with standard deviation 0.25, and the proposal distribution for *θ* used in the nested MCMC is a normal distribution, centred at the previous value and with standard deviation 10^–5^. The priors for both parameters are uniformly distributed within a compact domain. We set the shrink magnitude *n*_0_ = 2000 and precision parameter κ*p* = 4, *p* = 1,…,20. The SACut and naive SACut algorithms are processed in parallel on ten cores of Intel Xeon E7-8860 v3 CPU (2.2 GHz), and the (inherently serial) nested MCMC algorithm is processed on a single core. All algorithms were independently run 20 times, and the results are the averages across runs. Each run consists 5 × 10^4^ iterations. We retain only every tenth value after discarding the first 40% samples as burn-in.

To assess the performance of these algorithms, we compare their estimation of 𝔼(*θ*), lag-1 auto-correlation of samples, the Gelman-Rubin diagnostic statistic $\hat{R}$ (Gelman and Rubin 1992) and the average time needed for the whole run. The precision of the estimation of *θ* is measured by the mean square error (MSE) across its *d* (either 1 or 20) components. The convergence is evaluated by averaging the Gelman–Rubin diagnostic statistic of *d* components.

Results are shown in Table 1. The time required to run the nested MCMC algorithm increases as the length of the internal chain or dimension of *θ* increases, although the influence of dimension of *θ* is relatively small. In a low-dimensional case (*d* = 1), the time needed to run SACut and naive SACut is more than the time needed to run the WinBUGS algorithm and nested MCMC algorithm when the length of internal chain is less than 500, but both the MSE and the Gelman–Rubin statistic are lower when using the SACut algorithm. In particular, the bias of the WinBUGS algorithm is large. There is only trivial difference in bias between SACut and nested MCMC when *n*_int_ ≥ 1000, but SACut is significantly faster than nested MCMC. In the higher-dimensional case (*d* = 20), both SACut and naive SACut significantly outperform the WinBUGS and nested MCMC algorithm in terms of MSE. Although the difference between SACut and nested MCMC with *n*_int_ = 1000 is small, the Gelman–Rubin statistic of the nested MCMC is still larger than the threshold 1.2 suggested by Brooks and Gelman(1998). TheMCMCchains produced by the nested MCMC converge better and the bias is smaller when *n*_int_ ≥ 1500, but the SACut algorithm still outperforms it according to MSE and Gelman–Rubin statistic, and takes less time. It is also clear that nested MCMC samples show very strong auto-correlation for both cases and thinning may not efficiently solve this issue (Link and Eaton 2012); both SACut and naive SACut do not show any auto-correlation. We also note that the estimates provided by SACut and naive SACut are almost identical in practice. However, since the time needed for both algorithm is almost the same, providing the full approach with a more solid theoretical foundation is a valuable contribution to the computational statistics literature for the cut distribution.

Jacob et al. (2020) recently proposed an unbiased coupling algorithm which can sample from the cut distribution. It requires running coupled Markov chains where samples from each chain marginally target the same true distribution. The estimator is completely unbiased when two chains meet. Drawing samples from the cut distribution using the unbiased coupling algorithm typically involves two stages. In general, the first stage involves running coupled chains for *φ* until they meet. For each sampled *φ*, the second stage involves running another set of coupled chains for *θ* until they meet. Although the algorithm is unbiased, as illustrated in Sects. 4.2, 4.3 and the discussion of Jacob et al. (2020), the number of iterations for coupled chains is determined by meeting times, which can be very large especially when the dimension of the parameter is high. As a comparison, we apply the unbiased coupled algorithm on this example by using the R package “unbiasedmcmc” provided by Jacob et al. (2020). To simplify the implementation and computation of the unbiased coupling algorithm, we consider a simplified scenario with an informative conjugate prior for *φ*, meaning we can omit the first stage and instead directly draw5×10^4^ samples from *p*(*φ*|*Z*). This prior is normal with mean equal to the true value of *φ*. We then ran preliminary coupled chains for *θ* that target *p*(*θ*|*Y*, *φ*)given these samples of φ so as to sample the meeting times. Over the 5×10^4^ independent runs, the 95% and 99% quantiles of meeting times were 44 and 147, respectively, when *d* = 1. Although the majority of meeting times are, relatively, small, their 95% and 99% quantiles were 3525 and 5442, respectively, when *d* = 20. To ensure that the total number of iterations covers the majority of meeting times, following Jacob et al. (2020), we set the minimum number of iterations for each coupled chain to ten times the 95% quantile of meeting times. The algorithm was processed in parallel on the same ten cores as SACut, and the final result is shown in Table 1. Notably, unlike the nested MCMC algorithm, the computational time of the unbiased coupling algorithm increases significantly when the dimension of *θ* increases because it takes more time for coupled chains to couple in high-dimensional cases. In the low-dimensional case (*d* = 1), the unbiased coupling algorithm performs better according to all metrics. In the higher-dimensional case (*d* = 20), the unbiased coupling algorithm achieves similar MSE to the SACut algorithm, but it takes considerably more computation time than SACut, even though the unbiased coupling algorithm was been conducted under a simplified setting (i.e. no coupled chain for φ).

### 4.3 Epidemiological example

We now consider an epidemiological study of the relation between high-risk human papillomavirus (HPV) prevalence and cervical cancer incidence (Maucort-Boulch et al. 2008), which was previously discussed by Plummer (2015). In this study, age-stratified HPV prevalence data and cancer incidence data were collected from 13 cities. The model is divided into two modules. The first module concerns the number of people with HPV infection in city *i*, denoted as *Z_i_*, out of a sample of *N_i_* women:

$$Z_{i}\sim \text{Bin}\left(N_{i},\varphi_{i}\right).$$

The second module describes the relation between the number of cancer cases *Y_i_* from *T_i_* person-years and incidence which is assumed to be linked with *φ_i_* by a log linear relationship:

$$Y_{i}\sim \text{Poisson}\left(T_{i}\left(\exp\left(\theta_{1}+\theta_{2}\varphi_{i}\right)\right)\right).$$

The log-linear dose–response relationship is speculative, so we apply the cut algorithm to prevent the feedback from the second module to the estimation of *φi* (Plummer 2015).

**Fig. 6:**
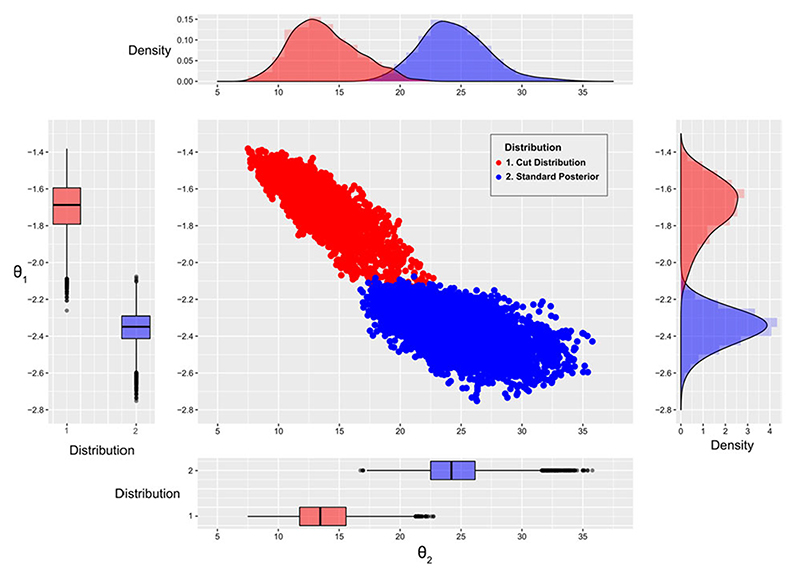
Comparison of the distribution of *θ*_1_ and *θ*_2_ drawn from the cut distribution (red) and standard Bayesian posterior (blue)

We apply the stochastic approximation cut algorithm and compare results with the standard Bayesian approach (i.e. without a cut). Both algorithms were run ten times independently, each with 1.4 × 10^5^ iterations. We set the shrink magnitude *n*_0_ = 20000 and precision parameter κ1 = 3 for *θ*_1_ and κ_2_ = 2 for *θ*_2_. We retain only every 100th value after discarding the first 4 × 10^4^ samples as burn-in. The pooled results of *θ* are shown in Fig. 6, highlighting the considerable effect of cutting feedback in this example. Our results are consistent with existing studies: specifically the scatter plot and density plot agree with Jacob et al. (2017) and Carmona and Nicholls (2020). Our results are also consistent with the results of nested MCMC algorithm when its internal chain length is largest (see Plummer (2015)). This again shows that the SACut algorithm provides similar estimates to the nested MCMC algorithm with a large internal chain length.

## 5 Conclusion

We have proposed a new algorithm for approximating the cut distribution that improves on the WinBUGS algorithm and approximate approaches in Plummer (2015). Our approach approximates the intractable marginal likelihood *p*(*Y*|*φ*) using stochastic approximation Monte Carlo (Liang et al. 2007). The algorithm avoids the weakness of approximate approaches that insert an “internal limit” into each iteration of the main Markov chain. Obviously, one can argue that approximate approaches can be revised by setting the length of the internal chain to the number of iterations, i.e.*n*_int_ = *n* so that the internal length diverges with *n*. However, since the sampling at each iteration is still not perfect and bias is inevitably introduced, the convergence of the main Markov chain remains unclear and the potential limit is not known. We proved convergence of the samples drawn by our algorithm and present the exact limit, though its convergence rate is not fully studied and needs further investigations. Although the bias is not completely removed by our algorithm, the degree of the bias is explicit in the sense that the shape of *p* ^κ^(*θ*|*Y*,*φ*) is known since the shape of *p*(*θ*|*Y*,*φ*) is normally obtainable given a fixed *φ*. Corollary 2 shows that the bias in our approach can be reduced by increasing the precision parameter κ. We proposed that κ be selected by comparing results across a range of choices; quantitative selection of this precision parameter still needs further study.

Existing approximate approaches (Plummer 2015) which need an infinitely long internal chain may be computationally slow, because the internal chain requires sequential calculation so parallelization is not possible. In contrast, thanks to the embarrassingly parallel calculation of (5), our algorithm can be more computationally efficient when multiple computer cores are available, although the per-iteration time of our algorithm decays as the Markov chain runs due to the increasing size of collection of auxiliary variables.

Lastly, while the adaptive exchange algorithm (Liang et al. 2016) is used for intractable normalizing problems when the normalizing function is an integral with respect to the observed data, it would be interesting to investigate the use of our algorithm for other problems involving a normalizing function that is an integral with respect to the unknown parameter. For example, our algorithm can be directly extended to sample from the recently developed semi-modular inference distribution (Carmona and Nicholls 2020) which generalizes the cut distribution.

## Supplementary Material

Supplementary Material

## Figures and Tables

**Table 1:** Mean squared error (MSE), lag-1 auto-correlation (in absolute value) |AC|, Gelman-Rubin statistic $\hat{R}$ , and clock time for the stochastic approximation cut (SACut) algorithm, naive SACut algorithm, WinBUGS algorithm, the nested MCMC algorithm (with varying internal chain length *n*_int_) and unbiased coupling algorithm

*d*	Algorithm	*n*_int_	MSE ×10^3^	|AC|	$\hat{R}$	Time (min)
1	SACut	–	0.112	0.019	1.00	311
	Naive SACut	–	0.114	0.016	1.00	308
	WinBUGS	1	355.280	0.999	308.78	1
	Nested MCMC	10	217.907	0.999	29.87	10
	Nested MCMC	200	0.158	0.997	1.74	182
	Nested MCMC	500	0.138	0.993	1.25	454
	Nested MCMC	1000	0.109	0.990	1.07	910
	Nested MCMC	1500	0.113	0.986	1.08	1349
	Nested MCMC	2000	0.118	0.981	1.05	1771
	Unbiased Coupling	–	0.114	0.012	1.01	22
20	SACut	–	1.42	0.009	1.00	1239
	Naive SACut	–	1.47	0.002	1.01	1219
	WinBUGS	1	16387.69	0.999	209.55	2
	Nested MCMC	10	12490.25	0.999	22.73	11
	Nested MCMC	200	249.18	0.999	2.38	259
	Nested MCMC	500	10.76	0.997	1.33	517
	Nested MCMC	1000	1.86	0.994	1.22	1010
	Nested MCMC	1500	1.69	0.991	1.19	1515
	Nested MCMC	2000	1.60	0.988	1.11	2058
	Unbiased Coupling	–	1.36	0.013	1.00	2030

All values are means across 20 independent runs

## Declarations

1
https://github.com/MathBilibili/Stochastic-approximation-cut-algorithm.

## References

[R1] Atchadé Y, Fort G, Moulines E, Priouret P, Barber D, Cemgil AT, Chiappa S (2011). Bayesian Time Series Models.

[R2] Bhattacharya A, Pati D, Yang Y (2019). Bayesian fractional posteriors. Ann Stat.

[R3] Blangiardo M, Hansell A, Richardson S (2011). A Bayesian model of time activity data to investigate health effect of air pollution in time series studies. Atmos Environ.

[R4] Brooks SP, Gelman A (1998). General methods for monitoring convergence of iterative simulations. J Comput Graph Stat.

[R5] Carmona CU, Nicholls GK (2020). Semi-modular inference: enhanced learning in multi-modular models by tempering the influence of components.

[R6] Fu JC, Wang L (2002). A random-discretization based Monte Carlo sampling method and its applications. Methodol Comput Appl Probab.

[R7] Gelman A, Rubin DB (1992). Inference from iterative simulation using multiple sequences. Stat Sci.

[R8] Gottardo R, Raftery AE (2008). Markov chain Monte Carlo with mixtures of mutually singular distributions. J Comput Graph Stat.

[R9] Haario H, Saksman E, Tamminen J (2001). An adaptive Metropolis algorithm. Bernoulli.

[R10] Huang B, Wu B, Barry M (2010). Geographically and temporally weighted regression for modeling spatio-temporal variation in house prices. Int J Geogr Inf Sci.

[R11] Jacob PE, Murray LM, Holmes CC, Robert CP (2017). Better together? Statistical learning in models made of modules. Preprint.

[R12] Jacob PE, O’Leary J, Atchadé YF (2020). Unbiased Markov chain Monte Carlo methods with couplings. J R Stat Soc B.

[R13] Liang F (2002). Dynamically weighted importance sampling in Monte Carlo computation. J Am Stat Assoc.

[R14] Liang F (2010). A double Metropolis-Hastings sampler for spatial models with intractable normalizing constants. J Stat Comput Simul.

[R15] Liang F, Liu C, Carroll RJ (2007). Stochastic approximation in Monte Carlo computation. J Am Stat Assoc.

[R16] Liang F, Jin IH, Song Q, Liu JS (2016). An adaptive exchange algorithm for sampling from distributions with intractable normalizing constants. J Am Stat Assoc.

[R17] Link WA, Eaton MJ (2012). On thinning of chains in MCMC. Methods Ecol Evol.

[R18] Liu F, Bayarri M, Berger J (2009). Modularization in Bayesian analysis, with emphasis on analysis of computer models. Bayesian Anal.

[R19] Liu Y, Lam K-F, Wu JT, Lam TT-Y (2018). Geographically weighted temporally correlated logistic regression model. Sci Rep.

[R20] Lunn D, Best N, Spiegelhalter D, Graham G, Neuenschwander B (2009a). Combining MCMC with ‘sequential’ PKPD modelling. J Pharmacokinet Phar.

[R21] Lunn D, Spiegelhalter D, Thomas A, Best N (2009b). The BUGS project: evolution, critique and future directions. Stat Med.

[R22] Malefaki S, Iliopoulos G (2009). Simulation from a target distribution based on discretization and weighting. Commun Stat Simul Comput.

[R23] Maucort-Boulch D, Franceschi S, Plummer M (2008). International correlation between human papillomavirus prevalence and cervical cancer incidence. Cancer Epidem Biomar.

[R241] McCandless LC, Douglas IJ, Evans SJ, Smeeth L (2010). Cutting feedback in Bayesian regression adjustment for the propensity score. Int J Biostat.

[R25] Meyn SP, Tweedie RL (1994). Computable bounds for geometric convergence rates of Markov chains. Ann Appl Probab.

[R26] Meyn S, Tweedie RL, Glynn PW (2009). Markov Chains and Stochastic Stability.

[R27] Miller JW, Dunson DB (2019). Robust Bayesian inference via coarsening. J Am Stat Assoc.

[R28] Møller J, Pettitt AN, Reeves R, Berthelsen KK (2006). An efficient Markov chain Monte Carlo method for distributions with intractable normalising constants. Biometrika.

[R29] Murray I, Ghahramani Z, MacKay DJC (2006). MCMC for doubly-intractable distributions.

[R30] Nakaya T, Fotheringham AS, Brunsdon C, Charlton M (2005). Geographically weighted Poisson regression for disease association mapping. Stat Med.

[R31] Park J, Haran M (2018). Bayesian inference in the presence of intractable normalizing functions. J Am Stat Assoc.

[R32] Plummer M (2015). Cuts in Bayesian graphical models. Stat Comput.

[R33] Roberts GO, Tweedie RL (1996). Geometric convergence and central limit theorems for multidimensional Hastings and Metropolis algorithms. Biometrika.

[R34] Walker SG (2013). Bayesian inference with misspecified models. J Stat Plan Inference.

[R35] Zigler CM (2016). The central role of Bayes’ theorem for joint estimation of causal effects and propensity scores. Am Stat.

