# Supplementary material for: Stochastic approximation cut algorithm for inference in modularized Bayesian models
Source: Stat Comput. Author manuscript; Available in PMC 2022 Feb 3. (PMC7612314; doi:10.1007/s11222-021-10070-2)
Supplement: Supplementary Material [file EMS140917-supplement-Supplementary_Material.pdf]

Stochastic Approximation Cut Algorithm for Inference in Modularized Bayesian Models

Yang Liu and Robert J.B. Goudie *

MRC Biostatistics Unit, University of Cambridge, Cambridge, UK

November 5, 2021

Supplementary Material for "Stochastic Approximation Cut Algorithm for Inference in Modularized Bayesian Model"

Submitted to *Statistics and Computing*

Appendices

A The construction of the auxiliary parameter set Φ_0

According to Liang et al. (2016), in order to have a good auxiliary parameter set Φ_0 so that the set of $p(\theta|Y,\varphi)$, $\varphi \in \Phi_0$, reasonably reflects the truth $p(\theta|Y,\varphi)$, where $\varphi \sim p(\varphi|Z)$, two conditions should be satisfied.

- Full representation: Let C_{Φ_0} be the convex hull constructed from Φ_0, then we require $\int_{C_{\Phi_0}} p(\varphi|Z)d\varphi \approx 1$. This ensures that the selected Φ_0 has fully represented the original domain of φ.

*E-mail address: yang.liu@mrc-bsu.cam.ac.uk (Yang Liu)

- Reasonable overlap: For neighbouring $\varphi_0^{(i)}$ and $\varphi_0^{(j)}$, we require $p(\theta|Y, \varphi_0^{(i)})$ and $p(\theta|Y, \varphi_0^{(j)})$ should have a reasonable overlap. Hence, the probability of accepting new proposal of $\varphi \in \Phi_0$ is reasonably large given a fixed θ for the auxiliary chain. This ensures that the auxiliary chain can mix well.

The grid Φ_0 is chosen by following the Max-Min procedure (Liang et al., 2016) and the purpose is to use this discrete set as a representation of the domain of $p(\varphi|Z)$. After we have decided the number of auxiliary parameters (that is m), the auxiliary parameter set Φ_0 is formed by selecting φ from a larger set $\Phi^{(M)} = \{\varphi^{(1)}, \cdots, \varphi^{(M)}\}$ (an arbitrary but substantially larger $M > m$) which are drawn from the marginal posterior $p(\varphi|Z)$ by any standard MCMC algorithm. Before starting the iterative process, we standardize all $\varphi_0^{(i)}, i = 1, \cdots, M$ (i.e, $\varphi_{new} = (\varphi - \varphi_{min})/(\varphi_{max} - \varphi_{min})$). We then add values to the grid using the following iterative process, initialised by randomly selecting a $\varphi_0^{(1)}$ as the first step. Then suppose at the kth step we have $\Phi_0^{(k)} = \{\varphi_0^{(1)}, \cdots, \varphi_0^{(k)}\}$. For each $\varphi \in \Phi^{(M)}$ that has not yet been selected, we calculate the minimum distance to the set $\Phi_0^{(k)}$ according to some pre-defined distance measure (e.g., Euclidean distance). This is the 'Min' process. We then find the φ that has not been selected but has the maximum distance to the set $\Phi_0^{(k)}$. This is the 'Max' process. We then add this particular φ into $\Phi_0^{(k)}$ to form $\Phi_0^{(k+1)} = \Phi_0^{(k)} \cup \{\varphi\}$.

Here we use a toy example to illustrate the procedure of selecting m. To make clear visualization of φ straightforward, we use the example derived from Section 4.1 in the main text but reduce the dimension of φ to 2.

We first draw a large number (M) of samples of φ from its marginal posterior $p(\varphi|Z)$ by a standard MCMC method and pool all samples together as a benchmark sample set. This step is feasible because $p(\varphi|Z)$ is a standard posterior distribution so there is no double intractability. If M is large enough, it is appropriate to regard the convex hull of the benchmark sample set as a good approximation of the true domain of $p(\varphi|Z)$.

Next, we select several candidates values of m. In this illustration, we simply select m from $\{10, 20, 50, 100, 500\}$. Given a selected value of m, we use the Max-Min procedure (Liang et al., 2016) to construct the $\Phi_0^{(m)}$, and calculate its corresponding convex hull $C_{\Phi_0^{(m)}}$ numerically using the R package *geometry*. See the attached Figure 1. It can be seen that the shape of the convex hull approximates the benchmark convex hull increasingly accurately as m increases.

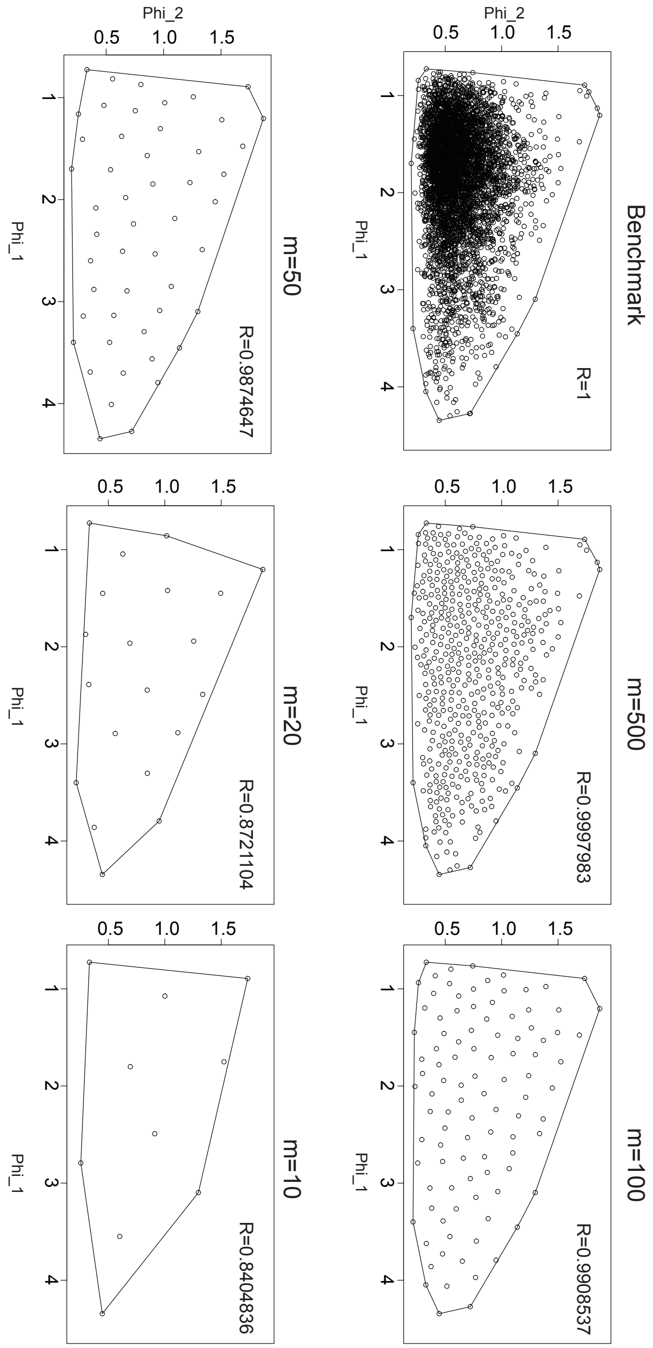

Figure 1: Convex hull C_{Φ_0} (indicated by the black circle) and samples of φ (indicated by dots) when m = 10, 20, 50, 100, 500 and Benchmark. The coverage ratio is shown on the upper right corner of each subplot.

Let $C_{\Phi_0^{(M)}}$ be the benchmark convex hull, and define the coverage ratio R_m by:

$$R_m = \frac{\int_{C_{\Phi_0^{(m)}}} p(\varphi|Z)d\varphi}{\int_{C_{\Phi_0^{(M)}}} p(\varphi|Z)d\varphi}, \tag{1}$$

where R_m can be numerically approximated using Monte Carlo. The m can be selected by choosing the smallest m that gives a high coverage ratio R_m (e.g., $R_m > 0.95$). In this example, since the the coverage ratio is > 0.98 we choose $m = 50$ to satisfy the first condition (Full representation).

We then check whether this value of m also satisfies the second condition (Reasonable overlap). This involves checking that the m different $p(\theta|Y, \varphi_0^{(i)})$, where $\varphi_0^{(i)} \in \Phi_0^{(m)}$, overlap sufficiently. To do this, for each $\varphi_0^{(i)}$, we draw samples of θ using a standard MCMC method and compare the empirical distribution of $\theta \sim p(\theta|Y, \varphi_0^{(i)})$ with the empirical distribution of $\theta \sim p(\theta|Y, \varphi_0^{(i1)})$ and $p(\theta|Y, \varphi_0^{(i2)})$, where $\varphi_0^{(i1)}$ and $\varphi_0^{(i2)}$ are the closest and second closest values in $\Phi_0^{(m)}$ to $\varphi_0^{(i)}$ in terms of Euclidean distance. This can be visually shown as a grouped box-plot in Figure 2. It is clear that the majority part (inter-quartile area) of the empirical distribution of θ given each $\varphi_0^{(i)} \in \Phi_0^{(m)}$ overlaps inter-quartile area given its neighbouring $\varphi_0^{(i1)}$ and $\varphi_0^{(i2)}$. Hence, it is appropriate to argue that $m = 50$ satisfies the second condition (Reasonable overlap).

In general, m grows with the dimension of φ. However, the exact relationship between them depends on the context of the real problem. Also, a large m is not a necessary condition for proposed theorems to be theoretically valid. This is because m is involved in the construction of the proposal distribution for the importance sampling procedure that forms the numerator and denominator of $P_n^*(\theta|Y, \varphi)$ and we could use any proposal distribution only if it has a correct domain, although a proposal distribution based on a smaller m could leads to a slow convergence of the auxiliary chain. In the special case when the marginal cut distribution $p(\theta|Y, \varphi)$ is not sensitive to the change of φ, a small m might be good enough. A larger m will bring fewer benefits when two conditions hold in practice. This differs significantly from the fact that we always require n to go to infinity. Hence, increasing m has a diminishing marginal utility after two conditions hold. Notwithstanding, when the dimension of φ increases, it will be more difficult to check whether two conditions hold because the numerical calculation of the convex hull will become extremely time-consuming and also checking for overlap visually will be infeasible. A more practical way could be simply checking whether the auxiliary chain can converge well given a reasonable time period by running some preliminary trials as suggested in Liang et al. (2016).

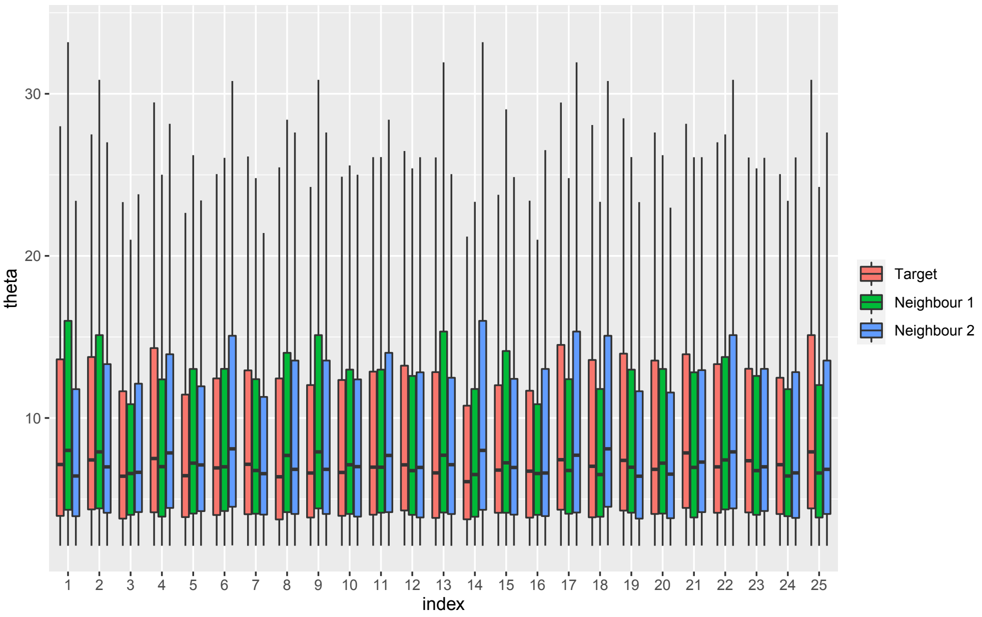

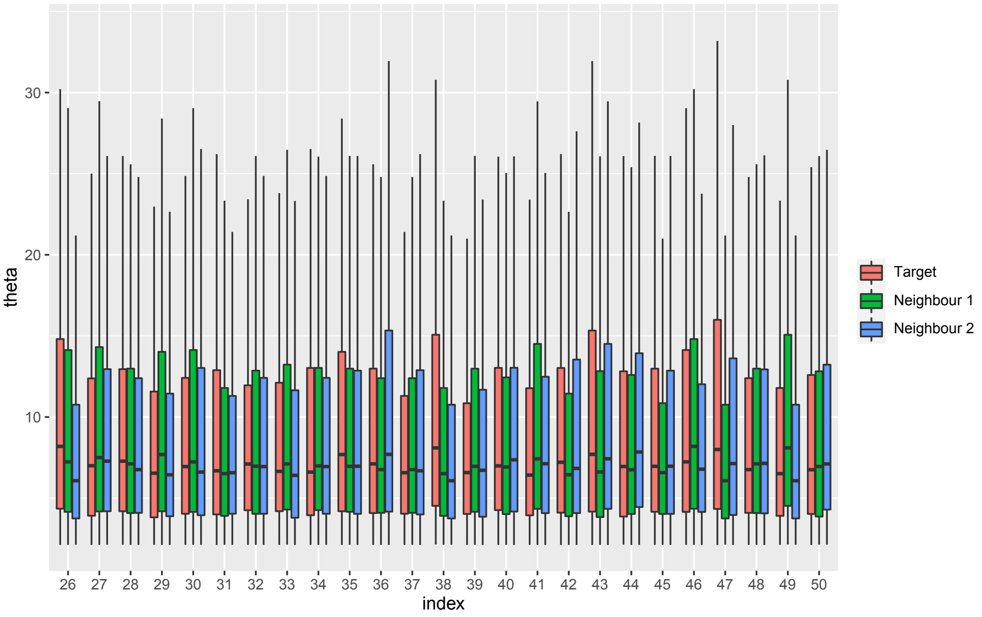

Figure 2: Boxplot of the empirical distribution of θ given φ (i.e., $p(\theta|Y, \varphi)$). For an arbitrary fixed index i, the red boxplot refers to $\varphi_0^{(i)}$. The green boxplot refers to $\varphi_0^{(i1)}$ (the closest neighbour). The blue boxplot refers to $\varphi_0^{(i2)}$ (the second closest neighbour).

B Construction of $P_n^*(\theta|Y,\varphi)$

Given a particular φ', measure $P_n^*(\theta|Y,\varphi)$ is actually a weighted average of $\mathbb{1}_{\{\theta\in\mathcal{B}\}}$. It is used to approximate $\int_{\mathcal{B}} p(Y|\theta,\varphi')p(\theta)d\theta/p(Y|\varphi')$, where numerator and denominator are separately approximated by dynamic importance sampling. The only difference is the domain of integration (i.e., $\mathcal{B}$ versus Θ). Here, we only show the numerator. We have

$$\int_{\mathcal{B}} p(Y|\theta,\varphi')p(\theta)d\theta \propto \mathbb{E}_\theta\left(\mathbb{1}_{\{\theta\in\mathcal{B}\}}\right), \textbf{ where } \theta \sim \frac{p(Y|\theta,\varphi')p(\theta)}{K(\varphi')},$$

where $K(\varphi')$ is a intractable normalizing constant of the target distribution. Hence, we cannot sample θ from $p(Y|\theta,\varphi')p(\theta)/K(\varphi')$. We have shown in the main text that, when iteration number j is large enough, we actually sample $(\tilde{\theta},\tilde{\varphi})$ from an iteration-specific proposal distribution

$$p_j(\tilde{\theta},\tilde{\varphi}) \propto \sum_{i=1}^{m} \frac{p(Y|\tilde{\theta},\varphi_0^{(i)})p(\tilde{\theta})}{\tilde{w}_{j-1}^{(i)}} \mathbb{1}_{\{\tilde{\varphi}=\varphi_0^{(i)}\}},\ \tilde{\theta}\in\Theta,\ \tilde{\varphi}\in\Phi_0.$$

Hence, we have

$$\begin{aligned}
&\mathbb{E}_\theta\left(\mathbb{1}_{\{\theta\in\mathcal{B}\}}\right) = \mathbb{E}_{\tilde{\theta},\tilde{\varphi}}\left(\mathbb{1}_{\{\tilde{\theta}\in\mathcal{B}\}}\frac{p(Y|\tilde{\theta},\tilde{\varphi}')p(\tilde{\theta})}{p_j(\tilde{\theta},\tilde{\varphi})K(\varphi')}\right)\\
&= \mathbb{E}_{\tilde{\theta},\tilde{\varphi}}\left\{\mathbb{1}_{\{\tilde{\theta}\in\mathcal{B}\}}\frac{p(Y|\tilde{\theta},\varphi')p(\tilde{\theta})}{K(\varphi')}\left(\sum_{i=1}^{m}\frac{\tilde{w}_{j-1}^{(i)}}{p(Y|\tilde{\theta},\varphi_0^{(i)})p(\tilde{\theta})}\mathbb{1}_{\{\varphi_0^{(i)}=\tilde{\varphi}\}}\right)\right\}, \textbf{ where } (\tilde{\theta},\tilde{\varphi}) \sim p_j(\tilde{\theta},\tilde{\varphi}).
\end{aligned}$$

The above expectation can be approximated by a step j single sample Monte Carlo estimator

$$\frac{1}{K(\varphi')}\sum_{i=1}^{m}\tilde{w}_{j-1}^{(i)}\frac{p(Y|\tilde{\theta}_j,\varphi')}{p(Y|\tilde{\theta}_j,\varphi_0^{(i)})}\mathbb{1}_{\{\tilde{\theta}_j\in\mathcal{B},\varphi_0^{(i)}=\tilde{\varphi}_j\}}, \textbf{ where } (\tilde{\theta}_j,\tilde{\varphi}_j) \sim p_j(\tilde{\theta},\tilde{\varphi}).$$

Note that, $K(\varphi')$ will be canceled out in the denominator and numerator of measure (5) so we can omit it. Now we have a total of n samples $\{(\tilde{\theta}_j,\tilde{\varphi}_j)\}_{j=1}^n$, we then add all single sample Monte Carlo estimators and calculate the average

$$\frac{1}{n}\sum_{j=1}^{n}\sum_{i=1}^{m}\tilde{w}_{j-1}^{(i)}\frac{p(Y|\tilde{\theta}_j,\varphi')}{p(Y|\tilde{\theta}_j,\varphi_0^{(i)})}\mathbb{1}_{\{\tilde{\theta}_j\in\mathcal{B},\varphi_0^{(i)}=\tilde{\varphi}_j\}}, \textbf{ where } (\tilde{\theta}_j,\tilde{\varphi}_j) \sim p_j(\tilde{\theta},\tilde{\varphi}).$$

Similarly, $1/n$ will be canceled out.

C Density Function Approximation by Simple Function

Here, we show how a density function f can be approximated by a simple function that is constant on a hypercube. We show that the degree of approximation can be easily controlled, and is dependent on the gradient of f. The use of a simple function to approximate a density function has been discussed previously (Fu and Wang, 2002; Malefaki and Iliopoulos, 2009), but here we use a different partition of the support of the function, determined by rounding to a user-specified number of decimal places.

For a compact set $\Psi \subset \mathbb{R}^d$ with dimension d, define a map $\mathcal{R}_\kappa : \Psi \to \Psi$ that rounds every element of $\psi \in \Psi$ to κ decimal places, where $\kappa \in \mathbb{Z}$, as $\mathcal{R}_\kappa(\psi) = \lfloor 10^\kappa \psi + 0.5 \rfloor / 10^\kappa$. Since Ψ is compact, $\mathcal{R}_\kappa(\Psi)$ is a finite set and we let R_κ denote its cardinality. We partition Ψ in terms of (partial) hypercubes Ψ_r whose centres $\psi_r \in \mathcal{R}_\kappa(\Psi)$ are the rounded elements of Ψ,

$$\Psi_r = \Psi \cap \{\psi : \|\psi - \psi_r\|_\infty \leq 5 \times 10^{-\kappa-1}\}, \ r = 1, ..., R_\kappa, \tag{2}$$

and the boundary set $\bar{\Psi}_\kappa$,

$$\bar{\Psi}_\kappa = \Psi \cap \left(\bigcup_{r=1}^{R_\kappa} \{\psi : \|\psi - \psi_r\|_\infty = 5 \times 10^{-\kappa-1}\} \right). \tag{3}$$

It is clear that $\bigcup_{r=1}^{R_\kappa} \Psi_r = \Psi$. Hence $\{\Psi_r \backslash \bar{\Psi}_\kappa\}_{r=1}^{R_\kappa}$ and $\bar{\Psi}_\kappa$ form a partition of Ψ.

Using this partition, we are able to construct a simple function density that approximates a density function. Let $\mathcal{C}$ be the set of all continuous and integrable probability density functions $f : \Psi \to \mathbb{R}$, and let $\mathcal{S}$ be the set of all simple functions $f : \Psi \to \mathbb{R}$. Define a map $\mathcal{S}_\kappa : \mathcal{C} \to \mathcal{S}$ for any $f \in \mathcal{C}$ as

$$\mathcal{S}_\kappa(f)(\psi) = \sum_{r=1}^{R_\kappa} \frac{1}{\mu(\Psi_r)} \int_{\Psi_r} f(\psi') d\psi' \mathbb{1}_{\{\psi \in \Psi_r\}}, \ \forall \psi \in \Psi.$$

The sets Ψ_r, $r = 1, ..., R_\kappa$, are the level sets of the simple function approximation, and the value $\mathcal{S}_\kappa(f)(\psi)$, $\psi \in \Psi \backslash \bar{\Psi}_\kappa$, is the (normalized) probability of a random variable with density f taking a value in Ψ_r, $r = 1, ..., R_\kappa$. Note that, when Ψ_r is a full hypercube, $\mu(\Psi_r) = 10^{-d\kappa}$; and if the set Ψ is known, then $\mu(\Psi_r)$ is obtainable for partial hypercubes. Figure 3 illustrates how this simple function approximates the truncated standard normal density function $f_{\text{norm}} : [-4, 4] \to \mathbb{R}$, when $\kappa = 0$ and $\kappa = 1$. Note that this is the optimal

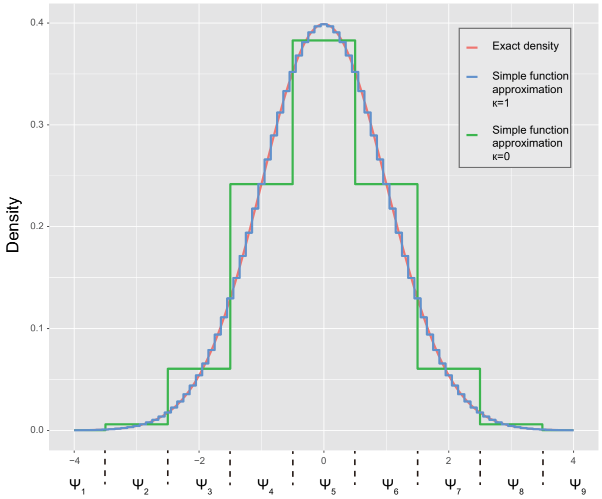

Figure 3: Simple function approximation of a truncated normal distribution. When $\kappa = 0$ the sets $\Psi_1 = [-4, -3.5]$, $\Psi_2 = [-3.5, -2.5]$,..., $\Psi_8 = [2.5, 3.5]$, $\Psi_9 = [3.5, 4]$ are the intervals partitioning $[-4, 4]$ and $\bar{\Psi}_0 = \{-3.5, -2.5, ..., 2.5, 3.5\}$.

simple function for the approximation in terms of Kullback-Leibler divergence (Malefaki and Iliopoulos, 2009).

Since $\mu(\bar{\Psi}_\kappa) = 0$, it is clear that

$$\int_\Psi \mathcal{S}_\kappa(f)(\psi)d\psi = \int_\Psi f(\psi)d\psi = 1.$$

Hence, $\mathcal{S}_\kappa(f)$ is a well-defined density function. We have the following theorem.

Theorem 1. *Given any continuous density function f,*

$$\mathcal{S}_\kappa(f) \xrightarrow{a.s.} f, \quad \text{as } \kappa \to \infty.$$

Proof. Let $\mathbb{Q}$ be the set of all rational numbers in $\mathbb{R}$ and hence $\mathbb{Q}^c$ is the set of all irrational numbers in $\mathbb{R}$. Let $\mathscr{E} = \mathbb{Q}^{cd} \cap \Psi$ and it is easy to see that $\mu(\mathscr{E}) = \mu(\Psi)$ since $\mu(\mathbb{Q}) = 0$. We first show that, $\forall \kappa < \infty$ and $\forall \psi \in \mathscr{E}$, we have $\psi \notin \bar{\Psi}_\kappa$.

Given a $\kappa < \infty$, every element of set $\mathcal{R}_\kappa(\Psi)$ is a d-dimensional rational vector. We also have that $5 \times 10^{-\kappa-1}$ is a rational number. Therefore, at least one element of d-dimensional vector ψ is a rational number if $\psi \in \bigcup_{r=1}^{R_\kappa} \{\psi : \|\psi - \psi_r\|_\infty = 5 \times 10^{-\kappa-1}\}$. Now $\forall \psi \in \mathscr{E}$,

because ψ is a d-dimensional irrational vector, $\psi \notin \bigcup_{r=1}^{R_\kappa}\{\psi : \|\psi - \psi_r\|_\infty = 5 \times 10^{-\kappa-1}\}$, and hence $\psi \notin \bar{\Psi}_\kappa$.

Now given a fixed $\kappa < \infty$, $\forall \psi \in \mathscr{E}$, since $\psi \notin \bar{\Psi}_\kappa$, ψ is always in the inner set of one of Ψ_r, $r = 1, ..., R_\kappa$. Re-write this Ψ_r as $\Psi_\psi^{(\kappa)}$. Since the set $\Psi_\psi^{(\kappa)}$ is compact and function f is continuous, we have $f_{\psi,min} = \min_{y \in \Psi_\psi^{(\kappa)}} f(y)$ and $f_{\psi,max} = \max_{y \in \Psi_\psi^{(\kappa)}} f(y)$. By the first mean value theorem, there is a $\psi^* \in \Psi_\psi^{(\kappa)}$ with $f_{\psi,min} \le f(\psi^*) \le f_{\psi,max}$, such that

$$\mathcal{S}_\kappa(f)(\psi) = \frac{1}{\mu(\Psi_\psi^{(\kappa)})} \int_{\Psi_\psi^{(\kappa)}} f(y)dy = f(\psi^*) \frac{1}{\mu(\Psi_\psi^{(\kappa)})} \int_{\Psi_\psi^{(\kappa)}} dy = f(\psi^*).$$

It is clear that, when κ increases, $\mu(\Psi_\psi^{(\kappa)})$ monotonically decreases since $\Psi_\psi^{(\kappa+1)} \subset \Psi_\psi^{(\kappa)}$ (i.e. a much smaller hypercube is formed). This leads to the fact that $(f_{\psi,max} - f_{\psi,min})$ monotonically decreases to 0. Hence, there is a N such that $\forall \kappa > N$, $(f_{\psi,max} - f_{\psi,min}) \le \varepsilon$. Then we have $\forall \kappa > N$,

$$|\mathcal{R}_\kappa^*(f)(\psi) - f(\psi)| = |f(\psi^*) - f(\psi)| \le (f_{\psi,max} - f_{\psi,min}) \le \varepsilon.$$

Hence,

$$\mathcal{S}_\kappa(f) \xrightarrow{\text{a.s.}} f, \quad \text{as } \kappa \to \infty.$$ □

When the density function f is also continuously differentiable, we can obtain the following result about the rate of convergence.

Corollary 1. *Given a density function f that is continuously differentiable, there exists a set $\mathscr{E} \subset \Psi$ with $\mu(\mathscr{E}) = \mu(\Psi)$ such that the local convergence holds:*

$$|\mathcal{S}_\kappa(f)(\psi) - f(\psi)| \le (\varepsilon(\psi, \kappa) + \|\nabla f(\psi)\|_2) \frac{\sqrt{d}}{10^\kappa}, \ \forall \psi \in \mathscr{E},$$

where $\varepsilon(\psi, \kappa) \to 0$ as $\kappa \to \infty$.

In addition, the global convergence holds:

$$\sup_{\psi \in \mathscr{E}} |\mathcal{S}_\kappa(f)(\psi) - f(\psi)| \le \sup_{\psi \in \Psi} \|\nabla f(\psi)\|_2 \frac{\sqrt{d}}{10^\kappa}.$$

Proof. Following the result of Theorem 1, for a given $\psi \in \mathscr{E}$, we have

$$|\mathcal{S}_\kappa(f)(\psi) - f(\psi)| \le (f_{\psi,max} - f_{\psi,min}).$$

Since f has a continuous gradient on a compact set, then by the mean value theorem we have:

$$(f_{\psi,max} - f_{\psi,min}) = |\langle \nabla f(y), (\psi_{max} - \psi_{min}) \rangle|.$$

where $\langle \cdot, \cdot \rangle$ means inner product, $f(\psi_{max}) = f_{\psi,max}$, $f(\psi_{min}) = f_{\psi,min}$, $y \in \Psi_{\psi}^{(\kappa)}$. By the Cauchy-Schwarz inequality, we have

$$|\langle \nabla f(y), (\psi_{max} - \psi_{min}) \rangle| \leq \|\nabla f(y)\|_2 \times \|(\psi_{max} - \psi_{min})\|_2$$

Now we prove the local convergence result. Since ∇f is continuous on the d-dimensional compact set Ψ, we can write

$$\varepsilon(\psi, \kappa) = \sup_{a,b \in \Psi_{\psi}^{(\kappa)}} \|\nabla f(a) - \nabla f(b)\|_2 .$$

Since $\mu(\Psi_{\psi}^{(\kappa)}) \to 0$, it is easy to check that $\varepsilon(\psi, \kappa) \to 0$ when $\kappa \to \infty$. Moreover, we have both ψ_{max} and ψ_{min} are in set $\Psi_{\psi}^{(\kappa)}$, and we have

$$\sup_{a,b \in \Psi_{\psi}^{(\kappa)}} \|(a-b)\|_2 = \sqrt{d 10^{-2\kappa}}.$$

Then by the triangle inequality, we have

$$\begin{aligned} \|\nabla f(y)\|_2 \times \|(\psi_{max} - \psi_{min})\|_2 &\leq (\|\nabla f(y) - \nabla f(\psi)\|_2 + \|\nabla f(\psi)\|_2) \frac{\sqrt{d}}{10^{\kappa}} \\ &\leq (\varepsilon(\psi, \kappa) + \|\nabla f(\psi)\|_2) \frac{\sqrt{d}}{10^{\kappa}}. \end{aligned}$$

and hence

$$|\mathcal{S}_{\kappa}(f)(\psi) - f(\psi)| \leq (\varepsilon(\psi, \kappa) + \|\nabla f(\psi)\|_2) \frac{\sqrt{d}}{10^{\kappa}}.$$

Now we prove the global convergence result. Since ∇f is continuous on compact set Ψ, then $\|\nabla f\|_2$ is bounded. We have

$$\|\nabla f(y)\|_2 \times \|(\psi_{max} - \psi_{min})\|_2 \leq \sup_{\psi \in \Psi} \|\nabla f(\psi)\|_2 \frac{\sqrt{d}}{10^{\kappa}}.$$

Therefore, we have

$$|\mathcal{S}_{\kappa}(f)(\psi) - f(\psi)| \leq \sup_{\psi \in \Psi} \|\nabla f(\psi)\|_2 \frac{\sqrt{d}}{10^{\kappa}}.$$

Note that, this means that $|\mathcal{S}_\kappa(f)(\psi) - f(\psi)|$ is uniformly bounded. Hence, it implies

$$\sup_{\psi \in \mathscr{E}} |\mathcal{S}_\kappa(f)(\psi) - f(\psi)| \leq \sup_{\psi \in \Psi} \|\nabla f(\psi)\|_2 \frac{\sqrt{d}}{10^\kappa}.$$

□

Corollary 1 shows that the rate of convergence of $\mathcal{S}_\kappa(f)$ to f is geometric. It states that, (a) for any $\psi \in \mathscr{E}$, the rate of convergence is locally controlled by its gradient $\|\nabla f(\psi)\|_2$; and (b) the rate of convergence is uniformly controlled by the upper bound of the gradient. Hence, as is intuitively expected, convergence is faster if the target function f has a smaller total variation on the set $\mathscr{E}$.

Remark 1. *When the scale of each component of $\psi \in \Psi$ is not same, a more complex partition can be formed by choosing component-specific precision parameters $\kappa = (\kappa_1, ..., \kappa_d)$. Denote $\circ$ as the Hadamard product and $10^{\pm\kappa} := (10^{\pm\kappa_1}, ..., 10^{\pm\kappa_d})$, we redefine*

$$\mathcal{R}_\kappa(\Psi) = \lfloor 10^\kappa \circ \psi + 0.5 \rfloor \circ 10^{-\kappa}.$$

We build a (partial) d-orthotope around $\psi_r \in \mathcal{R}_\kappa(\Psi)$

$$\Psi_r = \Psi \cap \{\psi : |\psi - \psi_r| \leqq 5 \times 10^{-\kappa-1}\}, \ r = 1, ..., R_\kappa.$$

We do not discuss this more complex partition but all results in this paper that are based on the basic partition in (2) and (3) can be easily extended to this more complex partition.

D Proofs of the Main Text

D.1 Proof of Lemma 1

We write the explicit form of $p^{(\kappa)}(\theta|Y, \varphi)$:

$$p^{(\kappa)}(\theta|Y, \varphi) = \mathcal{S}_\kappa(p(\cdot|Y, \varphi))(\theta) = \sum_{r=1}^{R_\kappa} \frac{1}{\mu(\Theta_r)} \int_{\Theta_r} p(\theta^*|Y, \varphi) d\theta^* \mathbb{1}_{\{\theta \in \Theta_r\}},$$

then we have:

$$
\begin{aligned}
&\sup_{\theta\in\Theta\setminus\bar{\Theta}_\kappa,\varphi\in\Phi} \left|p_n^{(\kappa)}(\theta|Y,\varphi) - p^{(\kappa)}(\theta|Y,\varphi)\right| \\
&= \sup_{\theta\in\Theta\setminus\bar{\Theta}_\kappa,\varphi\in\Phi} \left|\sum_{r=1}^{R_\kappa} \frac{1}{\mu(\Theta_r)} \left(W_n(\Theta_r|Y,\varphi) - \int_{\Theta_r} p(\theta^*|Y,\varphi)d\theta^*\right) \mathbb{1}_{\{\theta\in\Theta_r\}}\right| \\
&\le \sup_{\theta\in\Theta\setminus\bar{\Theta}_\kappa,\varphi\in\Phi} \sum_{r=1}^{R_\kappa} \frac{1}{\mu(\Theta_r)} \left|W_n(\Theta_r|Y,\varphi) - \int_{\Theta_r} p(\theta^*|Y,\varphi)d\theta^*\right| \mathbb{1}_{\{\theta\in\Theta_r\}} \\
&= \sup_{\varphi\in\Phi;1\le r\le R_\kappa} \frac{1}{\mu(\Theta_r)} \left|W_n(\Theta_r|Y,\varphi) - \int_{\Theta_r} p(\theta^*|Y,\varphi)d\theta^*\right|.
\end{aligned}
$$

Thus, using Equation 10 from the main text, it is clear that

$$
\lim_{n\to\infty} \sup_{\theta\in\Theta\setminus\bar{\Theta}_\kappa,\varphi\in\Phi} \left|p_n^{(\kappa)}(\theta|Y,\varphi) - p^{(\kappa)}(\theta|Y,\varphi)\right| = 0.
$$

Since $\mu(\bar{\Theta}_\kappa) = 0$, we are done.

D.2 Proof of Theorem 1

The theorem naturally holds when $n = 1$, we consider the case when $n \ge 2$. Since the dimension d and precision parameter κ are known and fixed, we suppose that the parameter space Θ is equally partitioned and the total number of d-orthotopes is R_κ and each orthotope is indexed as Θ_r, $r = 1, ..., R_\kappa$. Since we suppose that the auxiliary chain has converged before we start collecting auxiliary variable $\tilde{\theta}$, by equation 6 in the main text, we could write the probability of the original proposal distribution P_n^* taking a value in each partition component Θ_r as the integral with respect to the target distribution $p(\theta|Y,\varphi)$:

$$
W_\infty(\Theta_r|Y,\varphi) = \int_{\Theta_r} p(\theta|Y,\varphi)d\theta, \quad r = 1, ..., , R_\kappa.
$$

Now we define binary random variables I_r, $r = 1, ..., , R_\kappa$ as:

$$
I_r = \begin{cases} 1 & \text{if orthotope } r \text{ is never visited by auxiliary variables } \tilde{\theta}_i,\ i = 1, ..., n; \\ 0 & \text{otherwise,} \end{cases} \tag{4}
$$

We then have the expected number of orthotope visited is

$$\mathbb{E}\left(|\tilde{\Theta}_n^{(\kappa)}|\right) = \mathbb{E}\left(R_\kappa - \sum_{r=1}^{R_\kappa} I_r\right) = R_\kappa - \sum_{r=1}^{R_\kappa} \left(1 - W_\infty(\Theta_r|Y,\varphi)\right)^n.$$

By the method of the Lagrange multipliers, we write the Lagrange function as:

$$\mathcal{L}(W_\infty(\Theta_1|Y,\varphi), ..., W_\infty(\Theta_{R_\kappa}|Y,\varphi), \lambda) = R_\kappa - \sum_{r=1}^{R_\kappa} \left(1 - W_\infty(\Theta_r|Y,\varphi)\right)^n + \lambda\left(\sum_{r=1}^{R_\kappa} W_\infty(\Theta_r|Y,\varphi) - 1\right).$$

Conduct first order partial derivatives, we have

$$\frac{\partial \mathcal{L}}{\partial W_\infty(\Theta_r|Y,\varphi)} = -n\left(1 - W_\infty(\Theta_r|Y,\varphi)\right)^{n-1} + \lambda = 0, \quad r = 1, ..., R_\kappa;$$

$$\frac{\partial \mathcal{L}}{\partial \lambda} = \sum_{r=1}^{R_\kappa} W_\infty(\Theta_r|Y,\varphi) - 1 = 0.$$

These equations hold when $W_\infty(\Theta_r|Y,\varphi) = 1/R_\kappa$, $r = 1, ..., R_\kappa$. We now consider the second order derivatives, we have

$$\frac{\partial^2 \mathcal{L}}{\partial^2 W_\infty(\Theta_r|Y,\varphi)} = -n(n-1)\left(1 - W_\infty(\Theta_r|Y,\varphi)\right)^{n-2}, \quad r = 1, ..., R_\kappa;$$

$$\frac{\partial^2 \mathcal{L}}{\partial W_\infty(\Theta_r|Y,\varphi)\partial W_\infty(\Theta_t|Y,\varphi)} = 0, \quad r \neq t.$$

Hence the Hessian matrix is negative definite, $\mathbb{E}\left(|\tilde{\Theta}_n^{(\kappa)}|\right)$ achieves its maxima when $W_\infty(\Theta_r|Y,\varphi) = 1/R_\kappa$, $r = 1, ..., R_\kappa$. If we additionally require this to be held for any precision parameter κ, the target distribution $p(\theta|Y,\varphi)$ has to be uniform distribution.

D.3 Proof of Lemma 2

Given a $(\theta,\varphi) \in \Theta \times \Phi$, for any Borel set $\mathcal{B} = \mathcal{B}_\Theta \times \mathcal{B}_\Phi \subset \Theta \times \Phi$, define a signed measure D_n on $\Theta \times \Phi$ as

$$\begin{aligned}
D_n(\mathcal{B}|(\theta,\varphi)) &= \mathbf{T}_n^{(1)}\left(\mathcal{B}|(\theta,\varphi), \mathcal{G}_n\right) - \mathbf{U}^{(1)}(\mathcal{B}|(\theta,\varphi)) \\
&= \int_{\mathcal{B}_\Phi}\int_{\mathcal{B}_\Theta} \left(\alpha(\varphi'|\varphi)p^{(\kappa)}(\theta'|Y,\varphi')q(\varphi'|\varphi) - \alpha(\varphi'|\varphi)p_n^{(\kappa)}(\theta'|Y,\varphi')q(\varphi'|\varphi)\right) d\theta' d\varphi' \\
&= \int_{\mathcal{B}_\Phi}\left(\int_{\mathcal{B}_\Theta} \left(p^{(\kappa)}(\theta'|Y,\varphi') - p_n^{(\kappa)}(\theta'|Y,\varphi')\right) d\theta'\right) \alpha(\varphi'|\varphi)q(\varphi'|\varphi)d\varphi'.
\end{aligned}$$

Since $p(\varphi|Z)$ and $q(\varphi'|\varphi)$ are continuous on a compact set, then $\alpha(\varphi'|\varphi)$ and $q(\varphi'|\varphi)$ are bounded. Let $C = \sup_{\varphi'\in\Phi,\varphi\in\Phi} \alpha(\varphi'|\varphi)q(\varphi'|\varphi)$, we have

$$\begin{aligned}
&|D_n(\mathcal{B}|(\theta,\varphi))| \\
&= \left| \int_{\mathcal{B}_\Phi} \left(\int_{\mathcal{B}_\Theta \setminus \bar{\Theta}_\kappa} \left(p^{(\kappa)}(\theta'|Y,\varphi') - p_n^{(\kappa)}(\theta'|Y,\varphi') \right) d\theta' \right) \alpha(\varphi'|\varphi) q(\varphi'|\varphi) d\varphi' \right| \\
&\leq \int_{\mathcal{B}_\Phi} \sup_{\varphi^*\in\Phi} \left| \int_{\mathcal{B}_\Theta \setminus \bar{\Theta}_\kappa} \left(p^{(\kappa)}(\theta'|Y,\varphi^*) - p_n^{(\kappa)}(\theta'|Y,\varphi^*) \right) d\theta' \right| C d\varphi' \\
&\leq \mu(\Phi) C \int_{\mathcal{B}_\Theta \setminus \bar{\Theta}_\kappa} \sup_{\theta^*\in\Theta\setminus\bar{\Theta}_\kappa, \varphi^*\in\Phi} \left| p^{(\kappa)}(\theta^*|Y,\varphi^*) - p_n^{(\kappa)}(\theta^*|Y,\varphi^*) \right| d\theta' \\
&\leq \mu(\Phi)\mu(\Theta) C \sup_{\theta^*\in\Theta\setminus\bar{\Theta}_\kappa, \varphi^*\in\Phi} \left| p^{(\kappa)}(\theta^*|Y,\varphi^*) - p_n^{(\kappa)}(\theta^*|Y,\varphi^*) \right|.
\end{aligned}$$

The important fact here is that $|D_n(\mathcal{B}|(\theta,\varphi))|$ can be uniformly (with respect to θ, φ and Borel set $\mathcal{B}$) bounded by

$$\sup_{\theta^*\in\Theta\setminus\bar{\Theta}_\kappa, \varphi^*\in\Phi} \left| p^{(\kappa)}(\theta^*|Y,\varphi^*) - p_n^{(\kappa)}(\theta^*|Y,\varphi^*) \right|$$

up to a constant.

Given Lemma 1, we have that the density $p_n^{(\kappa)}$ converges almost surely to $p^{(\kappa)}$ and this convergence is uniformly on $\Theta \setminus \bar{\Theta}_\kappa \times \Phi$, and so we have

$$\lim_{n\to\infty} \sup_{\theta\in\Theta,\varphi\in\Phi} \|D_n(\cdot|(\theta,\varphi))\|_{TV} = 0.$$

Now by the triangle inequality, we have

$$\begin{aligned}
&\lim_{n\to\infty} \sup_{\theta\in\Theta,\varphi\in\Phi} \left\| \mathbf{T}_{n+1}^{(1)}\left(\cdot|(\theta,\varphi),\mathcal{G}_{n+1}\right) - \mathbf{T}_n^{(1)}\left(\cdot|(\theta,\varphi),\mathcal{G}_n\right) \right\|_{TV} \\
&\leq \lim_{n\to\infty} \sup_{\theta\in\Theta,\varphi\in\Phi} \|D_{n+1}(\cdot|(\theta,\varphi))\|_{TV} + \lim_{n\to\infty} \sup_{\theta\in\Theta,\varphi\in\Phi} \|D_n(\cdot|(\theta,\varphi))\|_{TV}.
\end{aligned}$$

It follows that:

$$\lim_{n\to\infty} \sup_{\theta\in\Theta,\varphi\in\Phi} \left\| \mathbf{T}_{n+1}^{(1)}\left(\cdot|(\theta,\varphi),\mathcal{G}_{n+1}\right) - \mathbf{T}_n^{(1)}\left(\cdot|(\theta,\varphi),\mathcal{G}_n\right) \right\|_{TV} = 0.$$

D.4 Proof of Lemma 3

Define a function $g : \Phi \to \mathbb{R}$ as

$$g(\varphi) = \min_{\theta \in \Theta} p_n^{(\kappa)}(\theta | Y, \varphi).$$

Since the support of $p_n^{(\kappa)}$ is Θ, we have $g(\varphi) > 0$, for all $\varphi \in \Phi$. In addition, since each element of $\mathscr{W}_n(\varphi)$ is a continuous function on the compact set Φ (see equation 5 and 9 in the main text), then $g(\varphi)$ is also a continuous function on Φ. Since Φ is compact, $g(\varphi)$ reaches its minima

$$\varepsilon = \min_{\varphi \in \Phi} g(\varphi).$$

Thus $p_n^{(\kappa)}(\theta | Y, \varphi) > \varepsilon$ for all $\theta \in \Theta$ and $\varphi \in \Phi$, and local positivity holds.

By the same reasoning, it is also true for the proposal distribution with density $p^{(\kappa)}$.

D.5 Necessary definitions

Definition 1. *Given any function $V : \Psi \to [1, \infty)$ and any signed measure $\mathcal{M}$ on Ψ, define the V-norm as*

$$\|\mathcal{M}\|_V = \sup_{|g| \leq V} \left| \int_\Psi g(\psi) \mathcal{M}(d\psi) \right|.$$

Definition 2. *For simplicity, for any function $f : \Psi \to \mathbb{R}$ and any measure $\mathcal{M}$ on Ψ, write*

$$\mathcal{M} f := \int_\Psi f(\psi) \mathcal{M}(d\psi).$$

Definition 3. *Given any two measures $\boldsymbol{M}_{(x)}(dz) := \boldsymbol{M}(dz|x)$, where $x \in \mathbb{X}$, and $\boldsymbol{N}_{(y)}(dx) := \boldsymbol{N}(dx|y)$ which concentrates on $\mathbb{X}$, for any Borel set $\mathcal{B}$, we write*

$$\boldsymbol{M}\boldsymbol{N}_{(y)}(\mathcal{B}) := \int_\mathcal{B} \int_\mathbb{X} \boldsymbol{M}_{(x)}(dz) \boldsymbol{N}_{(y)}(dx).$$

The definition can be extended to cases with more than two measures in a natural way.

D.6 Proof of Lemma 4

Given the filtration $\mathcal{G}_n$, the transition kernel $\mathbf{U}^{(1)}$ and $\mathbf{V}_n^{(1)}$ both admit an irreducible and aperiodic Markov chain by assumption. Therefore, to prove that transition kernel $\mathbf{T}_n^{(1)}$ also holds same property, it suffices to prove that for any $s \in \mathbb{N}$, $(\theta_0, \varphi_0) \in \Theta \times \Phi$, and Borel set $\mathcal{B} = \mathcal{B}_\Theta \times \mathcal{B}_\Phi \subset \Theta \times \Phi$ such that $\mathbf{V}_n^{(s)}(\mathcal{B}) > 0$, we have $\mathbf{T}_n^{(s)}(\mathcal{B}) > 0$. We prove this by mathematical induction.

Consider first when $s = 1$. We write $\alpha(\varphi'|\varphi) = \min(1, \beta(\varphi'|\varphi))$ where

$$\beta(\varphi'|\varphi) = \frac{p(\varphi'|Z)q(\varphi|\varphi')}{p(\varphi|Z)q(\varphi'|\varphi)},$$

and $\alpha_n((\theta', \varphi')|(\theta, \varphi)) = \min(1, \beta_n((\theta', \varphi')|(\theta, \varphi)))$, where

$$\beta_n((\theta', \varphi')|(\theta, \varphi)) = \frac{p^{(\kappa)}(\theta'|Y, \varphi')p(\varphi|Z)q(\varphi|\varphi')p_n^{(\kappa)}(\theta|Y, \varphi)}{p^{(\kappa)}(\theta|Y, \varphi)p(\varphi|Z)q(\varphi'|\varphi)p_n^{(\kappa)}(\theta'|Y, \varphi')},$$

and

$$r((\theta', \varphi'), (\theta, \varphi)) = \frac{\beta(\varphi'|\varphi)}{\beta_n((\theta', \varphi')|(\theta, \varphi))},$$

noting that both $p_n^{(\kappa)}$ and $p^{(k)}$ are bounded away from 0 and ∞. Now we denote

$$r^* = \min_{(\theta', \varphi'), (\theta, \varphi) \in \Theta \times \Phi} r((\theta', \varphi'), (\theta, \varphi)),$$

and it is easy to see that $r^* > 0$.

Now given any Borel set $\mathcal{B} = \mathcal{B}_\Theta \times \mathcal{B}_\Phi \subset \Theta \times \Phi$ and initial value $(\theta_0, \varphi_0) \in \Theta \times \Phi$, we have

$$\begin{aligned}
&\mathbf{T}_n^{(1)}\left(\mathcal{B}|(\theta_0, \varphi_0), \mathcal{G}_n\right) \\
&= \mathbf{T}_n^{(1)}\left(\mathcal{B} \setminus \{(\theta_0, \varphi_0)\}|(\theta_0, \varphi_0), \mathcal{G}_n\right) \\
&= \int_{\mathcal{B}} \alpha(\varphi|\varphi_0)p_n^{(\kappa)}(\theta|Y, \varphi)q(\varphi|\varphi_0)d\theta d\varphi \\
&= \int_{\mathcal{B}} \min\left\{1, r((\theta, \varphi), (\theta_0, \varphi_0))\beta_n((\theta, \varphi)|(\theta_0, \varphi_0))\right\} p_n^{(\kappa)}(\theta|Y, \varphi)q(\varphi|\varphi_0)d\theta d\varphi \\
&\geq \int_{\mathcal{B}} \min\left\{1, r((\theta, \varphi), (\theta_0, \varphi_0))\right\} \min\left\{1, \beta_n((\theta, \varphi)|(\theta_0, \varphi_0))\right\} p_n^{(\kappa)}(\theta|Y, \varphi)q(\varphi|\varphi_0)d\theta d\varphi \\
&\geq \min\left\{1, r^*\right\} \int_{\mathcal{B}} \alpha_n((\theta, \varphi)|(\theta_0, \varphi_0))p_n^{(\kappa)}(\theta|Y, \varphi)q(\varphi|\varphi_0)d\theta d\varphi
\end{aligned}$$

Since $\min\{1, r^*\} > 0$, we have

$$\mathbf{V}_n^{(1)}\left(\mathcal{B}|(\theta_0, \varphi_0), \mathcal{G}_n\right) > 0 \Rightarrow \mathbf{T}_n^{(1)}\left(\mathcal{B}|(\theta_0, \varphi_0), \mathcal{G}_n\right) > 0.$$

Thus, the induction assumption holds when $s = 1$.

Now assume that the induction assumption holds up to step $s = s^*$, i.e.

$$\mathbf{V}_n^{(s^*)}(\mathcal{B}) > 0 \Rightarrow \mathbf{T}_n^{(s^*)}(\mathcal{B}) > 0.$$

We need to show that it also holds at step $s = s^* + 1$. For an initial value (θ_0, φ_0), consider a Borel set $\mathcal{B}$ such that $\mathbf{V}_n^{(s^*+1)}(\mathcal{B}) > 0$. We proceed by contradiction. Suppose that

$$\mathbf{T}_n^{(s^*+1)}(\mathcal{B}) = \int_{\Theta\times\Phi} \mathbf{T}_n^{(1)}\left(\mathcal{B}|(\theta, \varphi), \mathcal{G}_n\right) \mathbf{T}_n^{(s^*)}(d\theta, d\varphi) = 0.$$

This implies that the function $\mathbf{T}_n^{(1)}\left(\mathcal{B}|\cdot, \mathcal{G}_n\right) = 0$ almost surely with respect to the measure $\mathbf{T}_n^{(s^*)}$. Because the induction assumption holds at step s^*, which means that any $\mathbf{V}_n^{(s^*)}$-measurable set of positive measure is a subset of a $\mathbf{T}_n^{(s^*)}$-measurable set of positive measure, we have that the function $\mathbf{T}_n^{(1)}\left(\mathcal{B}|\cdot, \mathcal{G}_n\right) = 0$ almost surely with respect to the measure $\mathbf{V}_n^{(s^*)}$. This further implies that the function $\mathbf{V}_n^{(1)}\left(\mathcal{B}|\cdot, \mathcal{G}_n\right) = 0$ almost surely with respect to the measure $\mathbf{V}_n^{(s^*)}$. It is clear that this contradicts the fact that $\mathbf{V}_n^{(s^*+1)}(\mathcal{B}) > 0$. Hence, we are done.

Given that $q(\varphi'|\varphi)$ and $p^{(\kappa)}(\theta'|Y, \varphi')$ satisfy the local positivity by Lemma 3, it is easy to check that

$$q((\theta', \varphi')|(\theta, \varphi)) = p^{(\kappa)}(\theta'|Y, \varphi')q(\varphi'|\varphi)$$

also satisfies local positivity. Hence, by Theorem 2.2 of Roberts and Tweedie (1996), since the target distribution is bounded away from 0 and ∞ on a compact set and the proposal distribution satisfies local positivity, the Partial Gibbs chain is irreducible and aperiodic, and every nonempty compact set is small. Moreover, $\Theta \times \Phi$ is a small set for the transition kernel $\mathbf{u}^{(1)}(\cdot|(\theta, \varphi))$, since it is compact. Hence, it is straightforward to verify that, for any $(\theta, \varphi) \in \Theta \times \Phi$ and Borel set $\mathcal{B} \subset \Theta \times \Phi$, there exists a $\delta > 0$ such that

$$\mathbf{U}^{(1)}(\mathcal{B}|(\theta, \varphi)) \geq \delta\mu(\mathcal{B}).$$

Since

$$q_n((\theta', \varphi')|(\theta, \varphi)) = p_n^{(\kappa)}(\theta'|Y, \varphi')q(\varphi'|\varphi)$$

also satisfies local positivity, following the proof of Theorem 2.2 in Roberts and Tweedie (1996) [1], one can show that, $\Theta \times \Phi$ is also a small set for the transition kernel $\mathbf{t}_n^{(1)}$. Let the "geometric drift function" $V(\theta, \varphi) \equiv 1$, there exists $\lambda < 1$ and $b < \infty$ such that

$$1 = \int_{\Theta \times \Phi} V(\theta^*, \varphi^*) \mathbf{T}_n^{(1)} \left((d\theta^*, d\varphi^*) | (\theta, \varphi), \mathcal{G}_n \right) \leq \lambda V(\theta, \varphi) + b \mathbb{1}_{\{(\theta, \varphi) \in \Theta \times \Phi\}}$$

then by Theorem 3.1 of Roberts and Tweedie (1996), for all $(\theta_0, \varphi_0) \in \Theta \times \Phi$, there exists a probability measure Π_n on $\Theta \times \Phi$ and constant $\rho < 1$ and $R < \infty$ such that for all $s = 1, 2, ...$ and all $(\theta_0, \varphi_0) \in \Theta \times \Phi$,

$$\left\| \mathbf{T}_n^{(s)} - \Pi_n \right\|_V \leq R \; V(\theta_0, \varphi_0) \rho^s.$$

Since $V = 1$, we have uniformly geometric convergence:

$$\lim_{s \to \infty} \sup_{(\theta_0, \varphi_0) \in \Theta \times \Phi} \left\| \mathbf{T}_n^{(s)} - \Pi_n \right\|_V = 0$$

In addition, for any $(\theta_0, \varphi_0) \in \Theta \times \Phi$,

$$0 \leq \left\| \mathbf{T}_n^{(s)}(\cdot) - \Pi_n(\cdot) \right\|_{TV} \leq \left\| \mathbf{T}_n^{(s)} - \Pi_n \right\|_V,$$

by the squeeze theorem, we have:

$$\lim_{s \to \infty} \sup_{(\theta_0, \varphi_0) \in \Theta \times \Phi} \left\| \mathbf{T}_n^{(s)}(\cdot) - \Pi_n(\cdot) \right\|_{TV} = 0.$$

Remark 2. *Following the fact that, for any $(\theta, \varphi) \in \Theta \times \Phi$ and Borel set $\mathcal{B} \subset \Theta \times \Phi$, there exists a $\delta > 0$ such that*

$$\boldsymbol{U}^{(1)}(\mathcal{B}|(\theta, \varphi)) \geq \delta \mu(\mathcal{B}).$$

following the proof of Lemma 2, we have:

$$\begin{aligned}
\boldsymbol{U}^{(1)}(\mathcal{B}|(\theta, \varphi)) &= \boldsymbol{U}^{(1)}(\mathcal{B}|(\theta, \varphi)) - \boldsymbol{T}_n^{(1)}(\mathcal{B}|(\theta, \varphi), \mathcal{G}_n) + \boldsymbol{T}_n^{(1)}(\mathcal{B}|(\theta, \varphi), \mathcal{G}_n) \\
&\leq \sup_{\theta \in \Theta, \varphi \in \Phi} \left| \boldsymbol{U}^{(1)}(\mathcal{B}|(\theta, \varphi)) - \boldsymbol{T}_n^{(1)}(\mathcal{B}|(\theta, \varphi), \mathcal{G}_n) \right| + \boldsymbol{T}_n^{(1)}(\mathcal{B}|(\theta, \varphi), \mathcal{G}_n) \\
&\leq C\mu(\mathcal{B}) \sup_{\theta^* \in \Theta \setminus \bar{\Theta}_\kappa, \varphi^* \in \Phi} \left| p^{(\kappa)}(\theta^*|Y, \varphi^*) - p_n^{(\kappa)}(\theta^*|Y, \varphi^*) \right| + \boldsymbol{T}_n^{(1)}(\mathcal{B}|(\theta, \varphi), \mathcal{G}_n).
\end{aligned}$$

[1] The difference is that there is an additional term, the ratio of $p_n^{(\kappa)}$ to $p^{(\kappa)}$, in our case. Since they are positive and bounded functions defined on $\Theta \times \Phi$, this ratio has a positive minimum on $\Theta \times \Phi$. Hence, the inequality in the original proof still holds.

where C is a constant. Therefore, for any $(\theta, \varphi) \in \Theta \times \Phi$ and Borel set $\mathcal{B} \subset \Theta \times \Phi$, we have

$$\boldsymbol{T}_n^{(1)}(\mathcal{B}|(\theta,\varphi),\mathcal{G}_n) \geq \left(\delta - C \sup_{\theta^* \in \Theta \backslash \bar{\Theta}_\kappa, \varphi^* \in \Phi} \left| p^{(\kappa)}(\theta^*|Y,\varphi^*) - p_n^{(\kappa)}(\theta^*|Y,\varphi^*) \right| \right) \mu(\mathcal{B}).$$

Note that, by Lemma 1, for any outcome ω in probability space Ω, we have

$$\sup_{\theta^* \in \Theta \backslash \bar{\Theta}_\kappa, \varphi^* \in \Phi} \left| p^{(\kappa)}(\theta^*|Y,\varphi^*) - p_n^{(\kappa)}(\theta^*|Y,\varphi^*) \right| \to 0, \text{ when } n \to \infty.$$

This is important. Since for any positive constant $a < \delta$, there exists a N such that for all $n > N$, we have

$$\boldsymbol{T}_n^{(1)}(\mathcal{B}|(\theta,\varphi),\mathcal{G}_n) \geq (\delta - a)\mu(\mathcal{B}).$$

Hence, a common and same lower bound is well defined on this outcome ω.

D.7 Proof of Lemma 5

For any initial value (θ_0, φ_0) and $s > 1$ and function $f : \Theta \times \Phi \to [-1, 1]$, write

$$\mathbf{T}_n^{(s)} f - P_{cut}^{(\kappa)} f = \mathbf{U}^{(s)} f - P_{cut}^{(\kappa)} f + \mathbf{T}_n^{(s)} f - \mathbf{U}^{(s)} f.$$

We first concentrate on the second term $\mathbf{T}_n^{(s)} f - \mathbf{U}^{(s)} f$, for any $1 \leq s_0 < s$, denote $\mathbf{U}^{(0)} = 1$ and $\mathbf{T}_n^{(0)} = 1$, we have, by a telescoping argument,

$$\begin{aligned}
&\left| \mathbf{T}_n^{(s)} f - \mathbf{U}^{(s)} f \right| \\
&\leq \left| \mathbf{T}_n^{(s)} f - \mathbf{T}_n^{(s_0)} f \right| + \left| \mathbf{T}_n^{(s_0)} f - \mathbf{U}^{(s_0)} f \right| + \left| \mathbf{U}^{(s)} f - \mathbf{U}^{(s_0)} f \right| \\
&= \left| \mathbf{T}_n^{(s)} f - \mathbf{T}_n^{(s_0)} f \right| + \left| \sum_{k=0}^{s_0-1} \left(\mathbf{U}^{(k)} \mathbf{T}_n^{(s_0-k)} f - \mathbf{U}^{(k+1)} \mathbf{T}_n^{(s_0-k-1)} f \right) \right| + \left| \mathbf{U}^{(s)} f - \mathbf{U}^{(s_0)} f \right| \\
&= \left| \mathbf{T}_n^{(s)} f - \mathbf{T}_n^{(s_0)} f \right| + \left| \sum_{k=0}^{s_0-1} \mathbf{U}^{(k)} \left(\mathbf{T}_n^{(1)} - \mathbf{U}^{(1)} \right) \mathbf{T}_n^{(s_0-k-1)} f \right| + \left| \mathbf{U}^{(s)} f - \mathbf{U}^{(s_0)} f \right|.
\end{aligned}$$

Note that, $\left(\mathbf{T}_n^{(1)} - \mathbf{U}^{(1)} \right)$ is the signed measure D_n defined in the proof of Lemma 2. By the result of Lemma 2, we have

$$\sup_{\theta \in \Theta, \varphi \in \Phi} \| D_n(\cdot|(\theta,\varphi)) \|_{TV} \xrightarrow{\text{a.s.}} 0,$$

on the probability space Ω. Then by Egorov's theorem, for any $e > 0$, there exists a set $E_1 \subset \Omega$ with $\mathbb{P}(E_1) > 1 - \frac{e}{2}$ such that $\sup_{\theta\in\Theta,\varphi\in\Phi} \|D_n(\cdot|(\theta,\varphi))\|_{TV}$ uniformly converges to 0 on E_1. Hence, for any $\epsilon > 0$, there exists a $N_1(\epsilon)$, such that for all $n > N_1(\epsilon)$, $\sup_{\theta\in\Theta,\varphi\in\Phi} \|D_n(\cdot|(\theta,\varphi))\|_{TV} \leq \epsilon$ on E_1. Then, since the remaining terms are bounded by 1, there exist a constant C such that

$$\left|\sum_{k=0}^{s_0-1} \mathbf{U}^{(k)} \left(\mathbf{T}_n^{(1)} - \mathbf{U}^{(1)}\right) \mathbf{T}_n^{(s_0-k-1)} f\right| \leq C s_0 \epsilon.$$

Now, following the same reasoning as Lemma 4 and Theorem 3.1 of Roberts and Tweedie (1996), $\mathbf{U}^{(s)}$ uniformly converges to $P_{cut}^{(\kappa)}$ in the sense of V-norm ($V \equiv 1$). Hence, for the same ϵ, there exists a $S_1(\epsilon)$ such that for any $s > s_0 > S_1(\epsilon)$,

$$\left|\mathbf{U}^{(s)} f - \mathbf{U}^{(s_0)} f\right| \leq \epsilon, \quad \left|\mathbf{U}^{(s)} f - P_{cut}^{(\kappa)} f\right| \leq \epsilon.$$

By Lemma 1, we have that $p_n^{(\kappa)}(\theta|Y,\varphi)$ converges to $p^{(\kappa)}(\theta|Y,\varphi)$ almost surely on probability space Ω. Then by Egorov's theorem, for same e, there exists a set $E_2 \subset \Omega$ with $\mathbb{P}(E_2) > 1 - \frac{e}{2}$ such that $p_n^{(\kappa)}(\theta|Y,\varphi)$ uniformly converges to $p^{(\kappa)}(\theta|Y,\varphi)$ on E_2. Hence on E_2, by the Remark of the proof of Lemma 4, for any Borel set $\mathcal{B} \subset \Theta \times \Phi$ and $(\theta,\varphi) \in \Theta \times \Phi$, there exists a N_2 such that for all $n > N_2$,

$$\mathbf{T}_n^{(1)} \left(\mathcal{B}|(\theta,\varphi),\mathcal{G}_n\right) \geq \frac{\delta}{2}\mu(\mathcal{B}).$$

By Theorem 2.3 of Meyn and Tweedie (1994), we have all $\mathbf{T}_n^{(1)} \left(\cdot|(\theta,\varphi),\mathcal{G}_n\right)$, when $n > N_2$, are uniformly ergodic in V-norm and have the same geometric convergence rate. Hence on E_2, there exists a $S_2(\epsilon)$, such that for all $s > s_0 > S_2(\epsilon)$ and $n > N_2$,

$$\left|\mathbf{T}_n^{(s)} f - \mathbf{T}_n^{(s_0)} f\right| \leq \epsilon.$$

Let $N(\epsilon) = \max(N_1(\epsilon), N_2)$ and $S(\epsilon) = \max(S_1(\epsilon), S_2(\epsilon))$. On set E_2, all convergences which involve $S_1(\epsilon)$ and $S_2(\epsilon)$ have geometric convergence rate. Thus, one can select a $S(\epsilon)$ such that $\epsilon S(\epsilon) \to 0$ when $\epsilon \to 0$.

Let $\varepsilon = (CS(\epsilon) + 3)\epsilon$ and set $E = E_1 \cap E_2$ with $\mathbb{P}(E) > 1 - e$. It is clear that $\varepsilon \to 0$ when $\epsilon \to 0$. We can conclude that, on set E, there exists $N(\epsilon)$ and $S(\epsilon)$ such that for any $n > N(\epsilon)$ and $s > S(\epsilon)$,

$$\left|\mathbf{T}_n^{(s)} f - P_{cut}^{(\kappa)} f\right| \leq \varepsilon.$$

Note that, for any Borel set $\mathcal{B} \subset \Theta \times \Phi$, we can let function f be an indicator function $\mathbb{1}_{\{x\in\mathcal{B}\}}$. Hence, for any initial value $(\theta_0, \varphi_0) \in \Theta \times \Phi$, and any $\varepsilon > 0$ and $e > 0$, there exists constants $S(\varepsilon) > 0$ and $N(\varepsilon) > 0$ such that

$$\mathbb{P}\left(\left\{P_n^{(\kappa)} : \left\|\mathbf{T}_n^{(s)}(\cdot) - P_{cut}^{(\kappa)}(\cdot)\right\|_{TV} \leq \varepsilon\right\}\right) > 1 - e.$$

for all $s > S(\varepsilon)$ and $n > N(\varepsilon)$.

D.8 Proof of Corollary 2

Given the result of global convergence in Corollary 1, and given a φ, there is a subset $\Theta^* \subset \Theta$ such that

$$\sup_{\theta\in\Theta^*} \left|p(\theta|Y,\varphi) - p^{(\kappa)}(\theta|Y,\varphi)\right| \leq \sup_{\theta\in\Theta^*} \|\nabla_\theta p(\theta|Y,\varphi)\|_2 \frac{\sqrt{d}}{10^\kappa},$$

where d is the dimension of θ. Following the proof of Lemma 1, we know that the construction of the set Θ^* is only related to the geometric shape of Θ, and it is not related to the function and thus not related to φ. Since p_{cut} is continuously differentiable, then $\nabla_{\theta,\varphi} p_{cut}(\theta, \varphi)$ is continuous. This further implies $\nabla_\theta p(\theta|Y, \varphi)$ is continuous with respect to θ and φ. Because Φ is compact, we have

$$\sup_{\theta\in\Theta^*,\varphi\in\Phi} \left|p(\theta|Y,\varphi) - p^{(\kappa)}(\theta|Y,\varphi)\right| \leq \sup_{\theta\in\Theta^*,\varphi\in\Phi} \|\nabla_\theta p(\theta|Y,\varphi)\|_2 \frac{\sqrt{d}}{10^\kappa} < \infty.$$

Now since $\mu(\Theta^*) = \mu(\Theta)$, we have the following bias term

$$\begin{aligned}
&\left|\int_{\Theta\times\Phi} f(\theta,\varphi) P_{cut}(d\theta, d\varphi) - \int_{\Theta\times\Phi} f(\theta,\varphi) P_{cut}^{(\kappa)}(d\theta, d\varphi)\right| \\
&= \left|\int_{\Theta^*\times\Phi} f(\theta,\varphi)\left(p(\theta|Y,\varphi) - p^{(\kappa)}(\theta|Y,\varphi)\right) p(\varphi|Z) d\theta d\varphi\right| \\
&\leq \int_{\Theta^*\times\Phi} f(\theta,\varphi) \left|p(\theta|Y,\varphi) - p^{(\kappa)}(\theta|Y,\varphi)\right| p(\varphi|Z) d\theta d\varphi \\
&\leq \sup_{\theta\in\Theta^*,\varphi\in\Phi} \left|p(\theta|Y,\varphi) - p^{(\kappa)}(\theta|Y,\varphi)\right| \int_{\Theta^*\times\Phi} f(\theta,\varphi) p(\varphi|Z) d\theta d\varphi \\
&\leq \sup_{\theta\in\Theta^*,\varphi\in\Phi} \|\nabla_\theta p(\theta|Y,\varphi)\|_2 \frac{\sqrt{d}}{10^\kappa} \left(\int_{\Theta^*\times\Phi} f(\theta,\varphi) p(\varphi|Z) d\theta d\varphi\right).
\end{aligned}$$

For any $\varepsilon > 0$, let

$$\sup_{\theta\in\Theta^*,\varphi\in\Phi} \|\nabla_\theta p(\theta|Y,\varphi)\|_2 \frac{\sqrt{d}}{10^\kappa}\left(\int_{\Theta^*\times\Phi} f(\theta,\varphi)p(\varphi|Z)d\theta d\varphi\right) = \frac{\varepsilon}{2},$$

let the solution of this equation be κ^*. We have the following bias term

$$\left|\int_{\Theta\times\Phi} f(\theta,\varphi)P_{cut}(d\theta,d\varphi) - \int_{\Theta\times\Phi} f(\theta,\varphi)P_{cut}^{(\kappa^*)}(d\theta,d\varphi)\right| \le \frac{\varepsilon}{2},$$

and this is always true in probability space Ω. Now by Theorem 2, for the same ε and κ^*, there exists a $N(\kappa^*,\varepsilon)$ such that for any $N > N(\kappa^*,\varepsilon)$, there is a set $E \subset \Omega$ with $\mathbb{P}(E) > 1 - e$ and on this set the error term satisfies

$$\left|\frac{1}{N}\sum_{n=1}^{N} f(\theta_n,\varphi_n) - \int_{\Theta\times\Phi} f(\theta,\varphi)P_{cut}^{(\kappa^*)}(d\theta,d\varphi)\right| \le \frac{\varepsilon}{2}.$$

Hence, combining the error term and bias term, on the set E we have

$$\begin{aligned}
&\left|\frac{1}{N}\sum_{n=1}^{N} f(\theta_n,\varphi_n) - \int_{\Theta\times\Phi} f(\theta,\varphi)P_{cut}(d\theta,d\varphi)\right| \\
&\le \left|\frac{1}{N}\sum_{n=1}^{N} f(\theta_n,\varphi_n) - \int_{\Theta\times\Phi} f(\theta,\varphi)P_{cut}^{(\kappa^*)}(d\theta,d\varphi)\right| + \left|\int_{\Theta\times\Phi} f(\theta,\varphi)P_{cut}(d\theta,d\varphi) - \int_{\Theta\times\Phi} f(\theta,\varphi)P_{cut}^{(\kappa^*)}(d\theta,d\varphi)\right| \le \varepsilon
\end{aligned}$$

Hence, we are done.

References

Fu, J. C. and Wang, L. (2002). A Random-Discretization Based Monte Carlo Sampling Method and its Applications. *Methodology and Computing in Applied Probability*, 4(1):5–25.

Liang, F., Jin, I. H., Song, Q., and Liu, J. S. (2016). An Adaptive Exchange Algorithm for Sampling from Distributions with Intractable Normalizing Constants. *Journal of the American Statistical Association*, 111(513):377–393.

Malefaki, S. and Iliopoulos, G. (2009). Simulation from a Target Distribution Based on Discretization and Weighting. *Communications in Statistics - Simulation and Computation*, 38(4):829–845.

Meyn, S. P. and Tweedie, R. L. (1994). Computable Bounds for Geometric Convergence Rates of Markov Chains. *The Annals of Applied Probability*, 4(4):981–1011.

Roberts, G. O. and Tweedie, R. L. (1996). Geometric Convergence and Central Limit Theorems for Multidimensional Hastings and Metropolis Algorithms. *Biometrika*, 83(1):95–110.
